# Structural basis for inhibition of the lipid flippase MurJ by phage lysis protein Sgl^PP7^ suggests convergent evolution with other Sgl peptides

**DOI:** 10.1371/journal.pbio.3004039

**Published:** 2026-10-27

**Authors:** Kaito Hosoda, Hidetaka Kohga, Sixian Wu, Hiroyuki Tanaka, Hideki Shigematsu, Ryoji Miyazaki, Min Fey Chek, Tomoya Tsukazaki

**Affiliations:** 1 Nara Institute of Science and Technology, Ikoma, Nara, Japan; 2 Diffraction and Scattering Division, Japan Synchrotron Radiation Research Institute, Sayo, Hyogo, Japan; University of Glasgow, UNITED KINGDOM OF GREAT BRITAIN AND NORTHERN IRELAND

## Abstract

Some bacteriophages encode lysis proteins that inhibit essential bacterial processes, and elucidating their mechanisms is valuable for developing antibacterial strategies against drug-resistant pathogens. We determined the cryo-EM structure of the complex between the essential *Escherichia coli* lipid II flippase MurJ and the phage lysis protein Sgl^PP7^. Structural, mutational, and molecular dynamics (MD) simulation analyses revealed that Sgl^PP7^ binds along the intramembrane cleft of MurJ and inserts its C-terminal tail into the internal cavity, stabilizing MurJ in an outward-facing conformation. Structural comparison with Lys^M^ (Sgl^M^) and Sgl^CJ3^ showed that these sequence-divergent Sgl proteins stabilize MurJ in the outward-facing conformation through distinct binding modes, consistent with the possibility of convergent evolution toward MurJ inhibition.

## Abstract 2

Bacteriophages encode lysis proteins that inhibit essential bacterial processes. This study solves the cryo-EM structure of the complex between the essential E. coli lipid II flippase MurJ and a phage lysis protein SglPP7, demonstrating that SglPP7 locks MurJ in an outward facing conformation.

## Introduction

Multidrug-resistant bacteria pose a major global public health challenge, and new antibacterial strategies are urgently needed. In gram-negative bacteria, peptidoglycan is important for maintaining cell shape and resistance to osmotic stress, and its biosynthesis pathway is a well-established target for antibiotics. Single-gene lysis (Sgl) proteins, encoded by single-stranded RNA and DNA phages and also known as “protein antibiotics,” induce host cell lysis by inhibiting essential cellular functions [1,2]. Recent metatranscriptomic analyses have greatly expanded the number of ssRNA phage genomes available in the NCBI database [3–5]. Although Sgl proteins had been identified only in representative ssRNA phages prior to this expansion, subsequent comprehensive analyses revealed diverse Sgl proteins, representing a rich source of potential “protein antibiotics.”

Phage-derived Sgl proteins primarily target essential factors of the peptidoglycan biosynthetic pathway. For example, protein E from the *microvirus* ΦX174 and protein A2 from the *Levivirus* Qβ have been shown to inhibit MraY and MurA, respectively [6–11]. Lys^M^, a 37-residue Sgl of *phage* M, induces cell lysis by targeting MurJ, the essential membrane protein that flips peptidoglycan precursor lipid II from the cytoplasm to the periplasm [12,13]. Furthermore, recent genetic screening suggested that Sgl^PP7^, a 55-residue Sgl of *Pseudomonas aeruginosa* phage PP7, also targets MurJ [14].

Structural studies have shown that MurJ, which consists of 14 transmembrane helices divided into an N-lobe (TM1–6), a C-lobe (TM7–12), and TM13–14, transports lipid II via alternating inward- and outward-facing conformations, similar to those of MATE transporters [15–18] (Fig 1A). Furthermore, cryo-EM analysis of the MurJ/Lys^M^ complex revealed that Lys^M^ wedges into the outward-facing conformation of MurJ, preventing the conformational changes as well as lipid II transport, which results in cell lysis [19]. In contrast to Lys^M^, Sgl^PP7^ is encoded at a distinct genomic position and shows no amino acid sequence similarity (Fig 1B and 1C). These findings raise the possibility that evolutionarily distinct phages independently acquired the ability to target MurJ.

**Fig 1:**
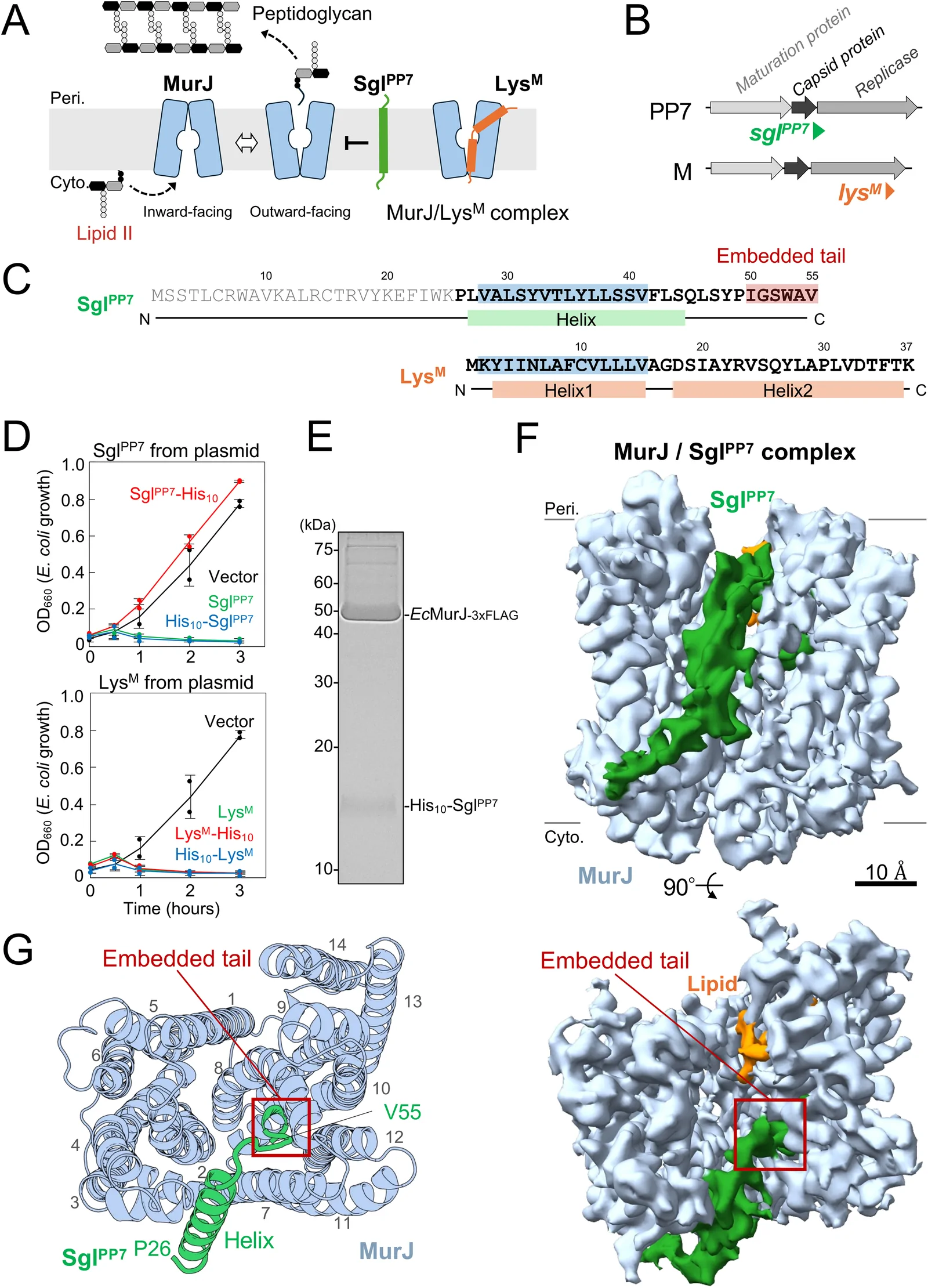
Distinct mode of MurJ inhibition by Sgl^PP7^ compared to Lys^M^. **(A)** Schematic illustration of the MurJ-mediated lipid II transport cycle and its inhibition by Sgl^PP7^ and Lys^M^. The inward-facing conformation captures lipid II from the cytoplasmic side, whereas the outward-facing conformation opens toward the periplasm, allowing lipid II to be released and incorporated into peptidoglycan. Sgl^PP7^ and Lys^M^ are shown as phage-derived lysis proteins that target MurJ. The previously determined structure of the MurJ/Lys^M^ complex revealed that Lys^M^ inhibits MurJ function by binding to its outward-facing conformation. **(B)** Genomic locations of the single-gene lysis protein genes *sgl*^*PP7*^ (green) in phage PP7 and *lys*^*M*^ (orange) in phage M. **(C)** Amino acid sequences and secondary structures of Sgl^PP7^ and Lys^M^. Disordered residues of Sgl^PP7^ are shown in gray. Regions in Sgl^PP7^ and Lys^M^ that occupy comparable structural positions are highlighted in blue. **(D)** Lytic activities of Sgl^PP7^ and Lys^M^. Growth curves of *E. coli* after overexpression of Sgl^PP7^ and Lys^M^ with or without a His_10_ tag from plasmids induced at 0 min. Error bars represent standard deviations from two biological replicates (*n* = 2), with individual data points overlaid. **(E)** SDS–PAGE analysis of the purified MurJ/Sgl^PP7^ complex. The gel was stained with Coomassie Brilliant Blue. **(F**, **G)** Cryo-EM structure of the MurJ/Sgl^PP7^ complex. **(F)** Top and side views of the sharpened cryo-EM map of the MurJ/Sgl^PP7^ complex (contour level: 0.03). MurJ, Sgl^PP7^, and lipid are shown in light blue, green, and orange, respectively. The scale bar represents 10 Å. **(G)** Top view of the MurJ/Sgl^PP7^ cartoon models, colored as in **(F)**. Transmembrane helices are numbered. The underlying data for (D) can be found in S1 Data. The original gel images of (E) can be found in S1 Raw Images.

Although another group reported the structure of a stable mutant MurJ/Sgl^PP7^ complex during the preparation of this manuscript, we independently determined the cryo-EM structure of a MurJ/Sgl^PP7^ complex reconstituted into lipid nanodiscs using full-length Sgl^PP7^. Based on mutational analysis and molecular dynamics (MD) simulations, we propose a model in which Sgl^PP7^ locks MurJ in an outward-facing conformation through interactions distinct from those of Lys^M^. These findings are consistent with the possibility that these sequence-divergent Sgl proteins independently acquired distinct strategies to inhibit MurJ.

## Results and discussion

### Overall structure of the MurJ/Sgl^PP7^ complex

We evaluated the toxicity of Sgl^PP7^ in *Escherichia coli* cells. The induction of untagged or N-terminal Met-His_10_-tagged Sgl^PP7^ caused cell lysis, whereas the C-terminal His_10_-tagged Sgl^PP7^ did not exhibit lytic activity (Fig 1D, upper). In contrast, Lys^M^, which shows little sequence similarity to Sgl^PP7^ (Fig 1C), retained lytic activity when fused with either an N- or C-terminal tag (Fig 1D, lower). These results indicate that although both Lys^M^ and Sgl^PP7^ are known to inhibit MurJ [14], Sgl^PP7^ may block MurJ through a mechanism distinct from that of Lys^M^. Therefore, the MurJ/Sgl^PP7^ complex was purified using N-terminally Met-His_10_-tagged Sgl^PP7^_1–55_(WT) and Met-Ala-*E. coli* MurJ_2–511_, reconstituted into nanodiscs with *E. coli* lipids, and its cryo-EM structure was determined at an overall resolution of 3.2 Å (Figs 1E–1G and S1; S1 Table). Recently, the structure of an N-terminally truncated, Met-Gly-His_6_-22× GS-tagged Sgl^PP7^_25–55_(S31A, S39A, S47A)/BRIL-MurJ_4–500_/Fab-nanobody complex was reported at a resolution of 3.70 Å [20]. Our structure was determined at a higher resolution using full-length Sgl^PP7^ reconstituted with MurJ in lipid nanodiscs (S2A–S2C Fig). Comparison of the two structures revealed a Cα root mean square deviation (RMSD) of 0.747 Å, indicating that the overall structures are nearly identical (S2D Fig). In both structures, Sgl^PP7^ is positioned along the intramembrane cleft of MurJ (Fig 1F). The modeled region of Sgl^PP7^ in each structure forms a single transmembrane helix followed by a short C-terminal tail that inserts deeply into a positively charged internal cavity on the C-lobe side of MurJ, formed by TM7, TM8, TM10, and TM12. We designate this feature as the “embedded tail” (Figs 1F, 1G, and S1F). In our structure, the N-terminal region of Sgl^PP7^ is modeled more extensively, beginning at residue 26 (S2C Fig). The structures suggest that Sgl^PP7^ stabilizes MurJ in the outward-facing conformation, arresting its transport cycle.

Notably, AlphaFold3 [21] failed to predict the experimentally observed positioning of Sgl^PP7^ on MurJ and showed low confidence in this region, highlighting the importance of the experimentally determined MurJ/Sgl^PP7^ complex structure (S3 Fig). Furthermore, all AlphaFold3-predicted MurJ models shown in S3 Fig. adopt an inward-facing conformation, in contrast to the outward-facing conformation observed in the experimental structures. This tendency may reflect the predominance of inward-facing MurJ structures among the structural data currently available in the Protein Data Bank (PDB). Thus, AlphaFold3 may preferentially predict the inward-facing state rather than the outward-facing conformation stabilized by Sgl^PP7^.

### Importance of N- and C-terminal regions of Sgl^PP7^

The cryo-EM map around the N-terminal region of Sgl^PP7^ (residues 1–25) was very poor and insufficient for reliable atomic modeling (S1D and S2C Figs). Therefore, residues 1–25 were not included in the atomic model, and the Sgl^PP7^ model starts at residue 26. To examine its functional importance, we generated a series of N-terminal truncation mutants of GFP-fused Sgl^PP7^ and evaluated their toxicity (S4 Fig). All truncation mutants retained lytic activity except for the GFP–Sgl^PP7^_22–55_, indicating that the minimal functional unit of Sgl^PP7^ resides in the region beginning around residue 22.

The C-terminal embedded tail (residues 50–55) inserts deeply into the positively charged interior of MurJ and likely plays an important role in this interaction. This structural feature is consistent with the loss of activity observed for the C-terminally His_10_-tagged Sgl^PP7^ (Fig 1D), indicating that the addition of a C-terminal tag sterically interferes with its insertion. Moreover, we addressed the importance of the embedded tail of Sgl^PP7^ by mutational analysis. Successive C-terminal truncations showed that retention of the sequence through residue 53 was required for lytic activity (Figs 2C, left; S5A). Next, we examined the lytic activity of several mutations at W53, which inserts deeply into the C-lobe and appears to play a key role in this interaction (Figs 2A–2C, middle; S5B). The W53A mutant retained full activity, W53G, W53C, and W53S showed slightly reduced activity, and W53K and W53R lost lytic activity. These results suggest that W53 is important for stabilizing the interaction with MurJ, although this effect may be partially compensated by neighboring residues, including residues 54–55. Additionally, we examined the same W53 variants in the truncated Sgl^PP7^ (1–53) mutant, which retains minimal activity, and all the variants showed weakened lytic activity (Figs 2C, right; S5C). These results suggest that the C-terminal region is an important determinant of Sgl^PP7^ function, with W53 serving as a key residue within this region.

**Fig 2:**
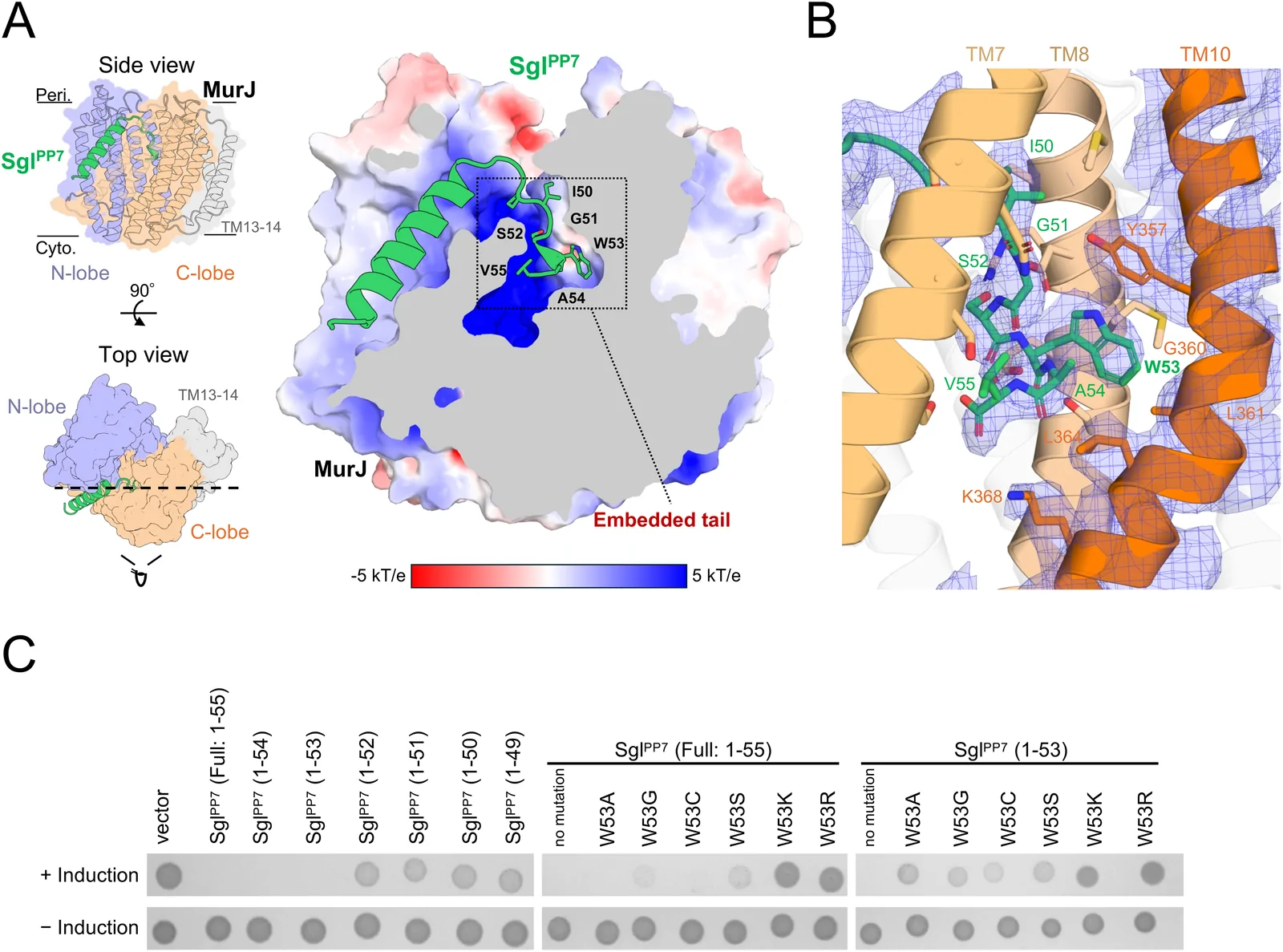
Embedded tail of Sgl^PP7^ inserted into the C-lobe cavity of outward-facing MurJ. **(A)** Cutaway views of the MurJ/Sgl^PP7^ complex along the dashed line. (Left) Side and top views of the MurJ/Sgl^PP7^ complex. The N-lobe, C-lobe, and TM13–14 of MurJ are shown in purple, orange, and gray, respectively. (Right) Cutaway view of MurJ is shown as an electrostatic potential surface, ranging from −5 kT/e (red) to +5 kT/e (blue). Residues 26–48 of Sgl^PP7^ are shown in cartoon representation, and residues 49–55 are shown in stick representation. **(B)** Close-up view of the embedded tail surrounded by TM7, TM8, and TM10 of MurJ. The C-lobe is viewed from the membrane, with the N-lobe omitted for clarity. The sharpened cryo-EM map of Sgl^PP7^ and TM10 in MurJ is shown at 4.4 σ. MurJ residues positioned within 4.5 Å of Sgl^PP7^ are shown as sticks. **(C)** Lytic activity of C-terminally truncated mutants (left) and W53 substitution mutants in full-length or truncated Sgl^PP7^ (1–53) (middle or right, respectively). *E. coli* cells harboring vector plasmids or plasmids encoding Sgl^PP7^ mutants were grown on agar plates with or without induction. Dark colonies indicate normal bacterial growth. The uncropped images of (C) can be found in S5 Fig.

### A lipid-like feature in the putative lipid II-binding groove of MurJ

In the cryo-EM map of our MurJ/Sgl^PP7^ complex, an unassigned lipid-like map feature is observed in the hydrophobic groove of MurJ, located between TM1, TM8, and TM14 (Figs 1F and 1G, and S6). A similar unmodeled lipid-like feature was also observed in the outward-facing crystal structure of *Thermosipho africanus* MurJ determined using lipidic cubic phase crystallization [16]. This groove was proposed to accommodate the hydrophobic undecaprenyl chain of lipid II.

The lipid-like map feature is located near F22, R24, and R270 in our structure (S6A and S6B Fig). R24 and R270 are conserved arginine residues located in the central cavity of MurJ and are essential for MurJ function [22–24]. D25 is structurally adjacent to R24 and R270 in the MurJ structures shown in S6C Fig. and has also been shown to be important for MurJ function [16]. Comparison of previously determined MurJ structures indicates that the relative positions of these residues change during the transport cycle (S6C Fig). In the inward-open structure of *T. africanus* MurJ, R255, which corresponds to R270 in *E. coli* MurJ, is oriented toward the central cavity. In the inward-occluded structure, R24 and R255 approach to within 2.9 Å, and this electrostatically unfavorable arrangement has been proposed to be stabilized through interactions with D25 and the negatively charged diphosphate moiety of lipid II [16]. In the outward-facing conformation, R255 is positioned away from R24 (S6C Fig). Thus, R24, D25, and R255/R270 undergo substantial rearrangement during the MurJ transport cycle, and the lipid-like map observed in our structure is located near these important residues, although it does not directly interact with them.

Furthermore, in vivo crosslinking analysis demonstrated that F22 on TM1 crosslinked with lipid II [25]. In addition, the crosslinking of lipid II was not significantly reduced in MurJ variants with substitutions at R24 or R270. These findings suggest that these residues may contribute to steps beyond the initial binding of lipid II, including conformational transitions associated with substrate recognition and transport.

In the MurJ/Sgl^PP7^ complex determined in this study, a lipid-like map was observed in this putative lipid II-binding region while MurJ was stabilized in an outward-facing conformation. However, the map does not contain features corresponding to the hydrophilic headgroup of lipid II, precluding identification of its molecular species. Therefore, these observations suggest only that a lipid-like molecule may be accommodated in the putative lipid II-binding region even in the Sgl^PP7^-bound state.

### Stabilization of MurJ in an outward-facing conformation by Sgl^PP7^

To evaluate how Sgl^PP7^ binding affects the structural dynamics of MurJ, we performed MD simulations in a membrane environment, both in the presence and absence of Sgl^PP7^ (Fig 3). In each of the three independent simulations for both conditions, the RMSD of the Cα atoms of MurJ increased over time. The RMSD values of the MurJ/Sgl^PP7^ complex were consistently lower than those of MurJ alone (Fig 3A). Comparison of the structures at 120 ns with the initial structures showed that MurJ maintained an outward-facing conformation both in the presence and absence of Sgl^PP7^ (Fig 3B). However, MurJ alone underwent larger structural fluctuations from the initial structures, whereas these fluctuations were suppressed in the MurJ/Sgl^PP7^ complex (Fig 3B).

**Fig 3:**
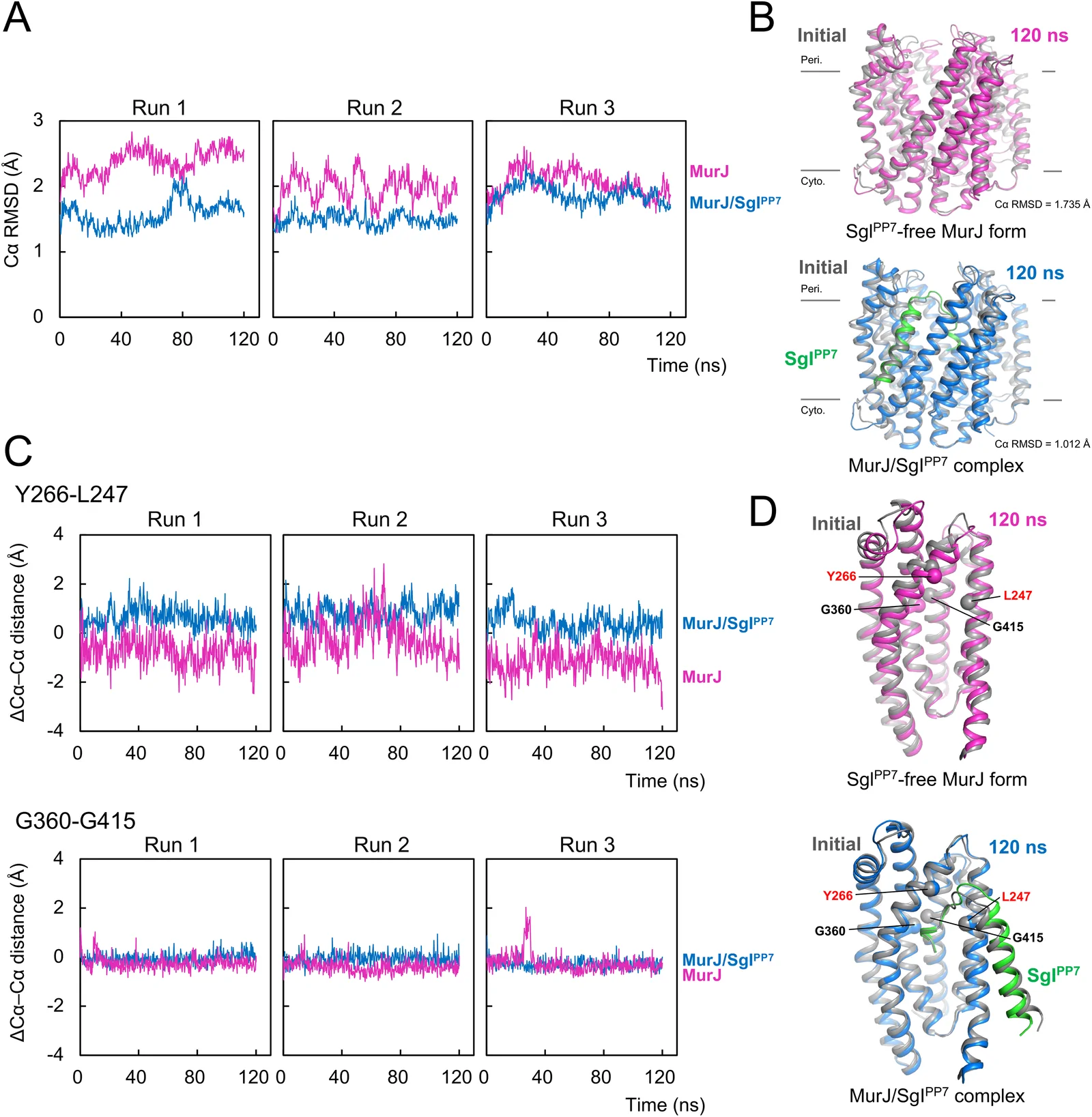
All-atom MD simulations of the MurJ/Sgl^PP7^ complex and MurJ in membrane. **(A)** Cα RMSD of the Sgl^PP7^-free MurJ (magenta) and MurJ in the MurJ/Sgl^PP7^ complex (blue). RMSD values are shown relative to the initial reference structure. Three independent simulations were performed using distinct POPE/POPG membrane environments. Detailed lipid compositions are described in Methods. **(B)** Overlay of the simulated structures at 120 ns with the corresponding initial reference structures (gray) in Run 1. The Sgl^PP7^-free MurJ, MurJ in the MurJ/Sgl^PP7^ complex, and Sgl^PP7^ are shown in magenta, blue, and green, respectively. **(C)** Changes in the Cα–Cα distances between Y266 on TM8 and L247 on TM7 (top) and between G360 on TM10 and G415 on TM12 (bottom), relative to their respective initial distances. Sgl^PP7^-free MurJ and MurJ in the MurJ/Sgl^PP7^ complex are shown in magenta and blue, respectively, as in **(A)**. **(D)** Close-up views of the C-lobe cavity showing superpositions of the structures at 120 ns with the corresponding initial structures from Run 1. The Cα atoms of Y266, L247, G360, and G415 are shown as spheres. The underlying data for (A and C) can be found in S1 Data.

During the simulations, the embedded tail of Sgl^PP7^ remained positioned within the internal cavity. To evaluate structural fluctuations around the embedded tail, we analyzed Cα–Cα distances of two residue pairs in transmembrane helices lining the C-lobe cavity of MurJ. Y266 on TM8 and L247 on TM7 flank the embedded tail in the MurJ/Sgl^PP7^ complex, whereas G360 on TM10 and G415 on TM12 are located deeper in the cavity and away from the embedded tail. In all three independent simulations, MurJ alone showed a tendency for the Cα–Cα distance between Y266 (TM8) and L247 (TM7) to decrease relative to the initial structure. In contrast, this distance was maintained in the MurJ/Sgl^PP7^ complex (Fig 3C and 3D). The distance between G360 and G415 remained largely unchanged under both conditions. Thus, these results support the idea that the C-terminal embedded tail of Sgl^PP7^, a structural feature distinct from Lys^M^, stabilizes MurJ in an outward-facing conformation.

### Structural comparison of the MurJ/Sgl^PP7^ complex with previously reported complexes

We compared the MurJ/Sgl^PP7^ complex with the MurJ/Lys^M^ complex. In both complexes, MurJ adopts an outward-facing conformation, with a Cα RMSD of 0.816 Å (Fig 4A). We further compared our MurJ/Sgl^PP7^ complex with recently reported MurJ/Lys^M^, MurJ/Sgl^PP7^, and MurJ/Sgl^CJ3^ structures [20]: the Cα RMSDs for the MurJ regions were 0.796, 0.747, and 0.704 Å, respectively. In all of these complexes, MurJ adopts a closely similar outward-facing conformation (S7 Fig). Lys^M^ consists of two helices (Helix 1 and Helix 2). Helix 1 is located peripherally near TM2 and TM7 (Fig 4A, right). In contrast, the Sgl^PP7^ TM helix is positioned closer to TM7 and adopts a different orientation relative to TM7 (Fig 4A). Moreover, the Sgl^PP7^ helix contacts a broader region along TM7 compared to Lys^M^ (Fig 4A, left). Comparison of the Sgl^PP7^ and Lys^M^ TM helices reveals distinct side-chain compositions at the MurJ interface, with non-overlapping residue sets, indicating different interaction modes (Fig 4B). In alanine-scanning mutagenesis, only F22A, L30A, and S39A of Sgl^PP7^ showed modest reductions in lytic activity (S9 Fig). In contrast, a recent study evaluated point mutations using a Δ2–24 minimal functional variant of Sgl^PP7^ as the background and reported that some substitutions, such as Y36 substitution, caused a marked loss of function [20]. Together, these results suggest that the Sgl^PP7^ TM helix does not rely on a few specific residue interactions but is instead supported by distributed, weak interactions across a broad contact surface. We evaluated the sensitivity of Lys^M^-resistant MurJ mutants carrying substitutions at the Lys^M^-binding interface to Sgl^PP7^. Under Sgl^PP7^ induction, only the Q244P mutant was able to grow, whereas all other mutants remained sensitive to Sgl^PP7^ (Fig 4C). These results also support that Sgl^PP7^ and Lys^M^ inhibit MurJ through distinct binding interfaces.

**Fig 4:**
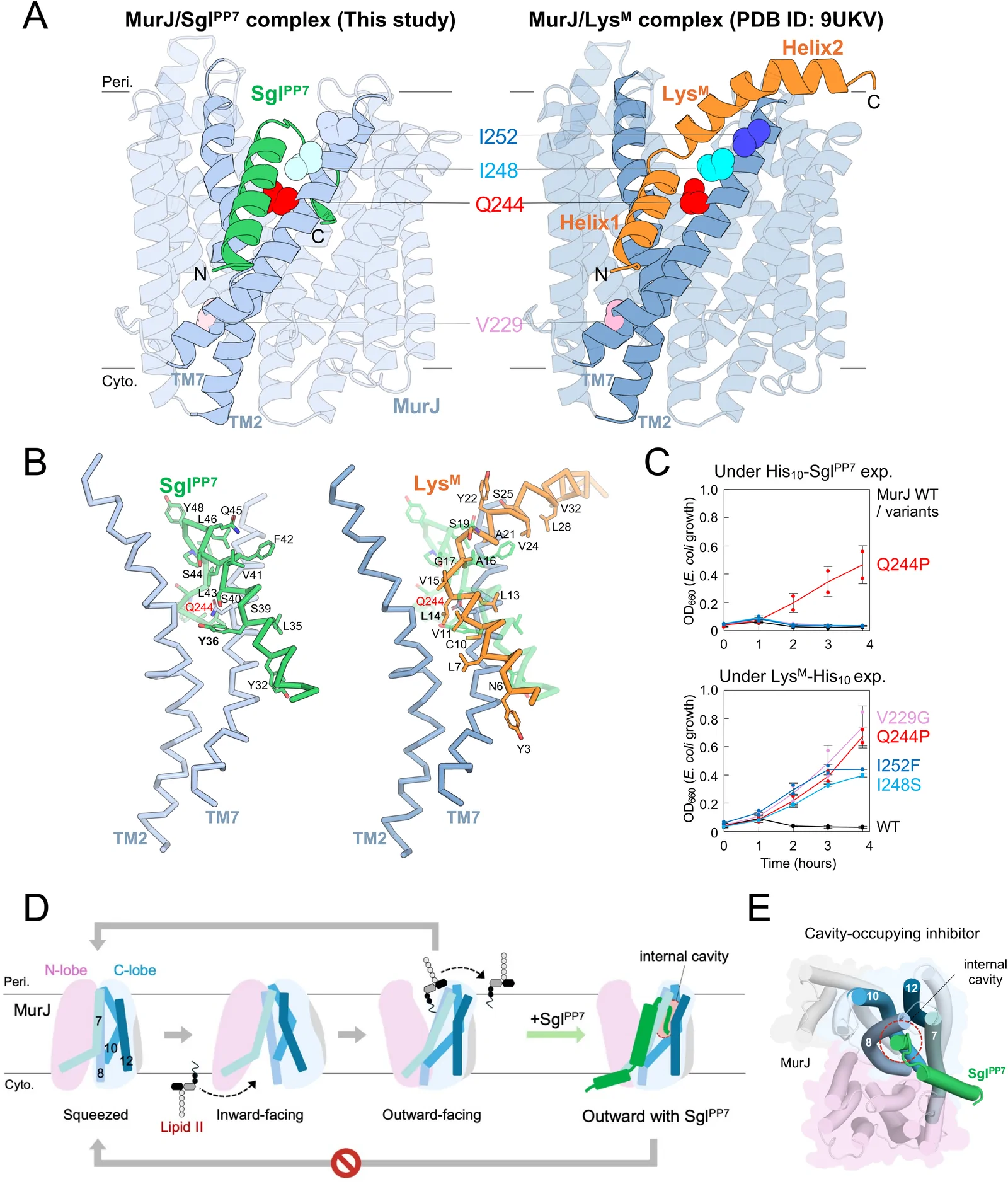
Comparison of the MurJ/Sgl^PP7^ and MurJ/Lys^M^ complexes. **(A)** Comparison of the binding modes of Sgl^PP7^ and Lys^M^ on outward-facing MurJ. The MurJ/Sgl^PP7^ complex (PDB: 9X2N) and the MurJ/Lys^M^ complex (PDB: 9UKV) are shown as cartoon models. MurJ and Sgl^PP7^ in the MurJ/Sgl^PP7^ complex are colored as in Fig 1G. MurJ and Lys^M^ in the MurJ/Lys^M^ complex are shown in deeper blue and orange, respectively. Positions of previously reported Lys^M^-resistance mutations [13] are mapped onto the MurJ/Sgl^PP7^ and MurJ/Lys^M^ complexes. **(B)** Interactions of Sgl^PP7^ and Lys^M^ with TM2 and TM7 of MurJ. TM2 and TM7 of MurJ and Sgls are shown as ribbon models, and contacting side chains are shown as sticks. **(C)** Activities of Sgl^PP7^ against Lys^M^-resistant MurJ mutants. Growth curves of *E. coli* expressing Lys^M^-resistant MurJ under Sgl^PP7^ or Lys^M^ induction (induction at 0 min, top). Error bars represent standard deviations from two biological replicates (*n* = 2), with individual data points overlaid. **(D–E)** Working model for Sgl^PP7^-mediated inhibition of MurJ. **(D)** Proposed model for Sgl^PP7^-mediated inhibition of the MurJ transport cycle. MurJ is proposed to cycle through squeezed, inward-facing, and outward-facing conformations during lipid II transport. In this process, lipid II is captured at the cytoplasmic side and released into the periplasm. Sgl^PP7^ inserts its C-terminal embedded tail into the internal cavity of the C-lobe, locking MurJ in the outward-facing conformation and blocking the lipid II transport cycle. **(E)** Binding mode of Sgl^PP7^ on MurJ. The MurJ/Sgl^PP7^ complex (PDB: 9X2N, this study) is shown as cylinder model. Sgl^PP7^ acts as a cavity-occupying inhibitor. The underlying data for (C) can be found in S1 Data.

We next examined the structural similarities and differences among Sgl^PP7^, Lys^M^, and Sgl^CJ3^ in detail (S7 and S8 Figs). Despite their highly divergent amino acid sequences (S7C Fig), the transmembrane helices of all three Sgl proteins are located at the interface between the N- and C-lobes of MurJ, particularly near TM2 and TM7. The transmembrane helix of Sgl^CJ3^ adopts a position and orientation similar to those of Sgl^PP7^ (S7B Fig), but distinct from that of Lys^M^. Furthermore, the side chains of Y36 of Sgl^PP7^, L14 of Lys^M^, and Y76 of Sgl^CJ3^ insert into a similar pocket on MurJ (Figs 4B and S8A). Y36 of Sgl^PP7^ and Y76 of Sgl^CJ3^ are located in similar positions on their helices, whereas L14 of Lys^M^, which is shifted by approximately one helical turn relative to these positions, inserts into a similar pocket. Consistent with this structural observation, substitution of Y76 markedly reduced the lytic activity of Sgl^CJ3^, supporting the functional importance of this residue in MurJ inhibition [20]. These structural and biochemical findings indicate that, despite differences in their sequences and transmembrane-helix arrangements, the three Sgl proteins target a similar structural region of MurJ.

In contrast, the C-terminal regions of the three Sgl proteins adopt markedly distinct arrangements on MurJ and interact with different regions of MurJ (S8B and S8C Fig). In Sgl^PP7^, the C-terminal embedded tail occupies the internal cavity of the C-lobe of MurJ and extends deeply into it (Figs 2 and S8C). In contrast, the C-terminal Helix 2 of Lys^M^ is positioned along the membrane surface and interacts with the external surface of MurJ (Figs 4A and S7). In Sgl^CJ3^, the transmembrane helix broadly occupies the membrane-embedded cleft formed by TM2 and TM7/TM8, whereas only part of the C-terminal region extends into the cavity. Thus, the three Sgl proteins interact with different regions of MurJ through their C-terminal regions and stabilize MurJ in the outward-facing conformation through distinct binding modes characterized as cavity-occupying, surface-binding, and cleft-binding for Sgl^PP7^, Lys^M^, and Sgl^CJ3^, respectively.

Similarly, phages λ, T7, T5, and P1 encode the small FtsZ inhibitors Kil, Gp0.4, Hdi, and Icd, respectively [26–29]. Despite having no apparent amino acid sequence similarity, these proteins all target the essential cell-division protein FtsZ. For these FtsZ inhibitors, structures of their complexes with FtsZ are not currently available, and it remains unclear whether they inhibit FtsZ through common or distinct mechanisms. In contrast, Sgl^PP7^, Lys^M^, and Sgl^CJ3^ provide a structurally characterized example of how sequence-divergent phage proteins inhibit the same essential bacterial protein through distinct binding modes.

### Sgl^PP7^ working model

Based on the MurJ/Sgl^PP7^ complex structure determined in this study, we propose a model for how Sgl^PP7^ inhibits the MurJ transport cycle (Fig 4D). During lipid II transport, MurJ undergoes conformational transitions from the inward-facing conformation to the outward-facing conformation and then returns to the inward-facing conformation via the squeezed conformation. Sgl^PP7^ forms a broad contact surface near TM7 of MurJ and inserts its C-terminal embedded tail deeply into the internal cavity of the C-lobe, locking MurJ in the outward-facing conformation (Fig 4E). In addition, a lipid-like map was observed in the putative lipid II-binding region of the MurJ/Sgl^PP7^ complex. These observations suggest that Sgl^PP7^ prevents outward-facing MurJ from undergoing subsequent conformational transitions. As a result, the lipid II transport cycle is blocked.

In this study, we elucidated how Sgl^PP7^ inhibits the MurJ transport cycle by forming a broad interaction interface near TM7 and inserting its C-terminal tail deeply into the internal cavity of the C-lobe, thereby stabilizing MurJ in the outward-facing conformation. Structural comparison of Sgl^PP7^, Lys^M^, and Sgl^CJ3^ further showed that, despite their highly divergent amino acid sequences and overall binding modes to MurJ, these Sgl proteins target a similar structural region of MurJ and stabilize the outward-facing conformation. Nevertheless, their binding modes are markedly distinct: cavity-occupying for Sgl^PP7^, surface-binding for Lys^M^, and cleft-binding for Sgl^CJ3^. Thus, these phage-derived Sgl proteins with no apparent amino acid sequence similarity and encoded at different genomic locations appear to have independently acquired distinct molecular strategies to inhibit the MurJ transport cycle. Their shared ability to stabilize MurJ in the outward-facing conformation is consistent with the possibility of convergent evolution toward MurJ inhibition.

## Methods

### Bacterial strains and plasmids

The *E. coli* strains and plasmids used in this study are listed in S2 Table. Details of the plasmids used are described in the Plasmid Construction section.

### Plasmid construction

A DNA fragment containing the *sgl*^*PP7*^ gene, which was codon-optimized for *E. coli*, was synthesized de novo (Integrated DNA Technologies, IDT). The *sgl*^*PP7*^ fragment was cloned into XbaI-digested pBAD33 [30] using In-Fusion HD Cloning Kit (Takara Bio). The resulting plasmid was designated pKG382. For pKH32 (pBAD18 [30] encoding *his*_*10*_*-sgl*^*PP7*^), the *his*_*10*_*-sgl*^*PP7*^ fragment was PCR-amplified from pKG382. The PCR product was cloned into inverse PCR-linearized pBAD18 using In-Fusion HD Cloning Kit (Takara Bio). Mutant plasmids were constructed using site-directed mutagenesis.

### Media and bacterial cultures

*E. coli* cells were grown at 37 °C in LB medium (Nacalai Tesque). Ampicillin (Amp) (100 µg/mL), chloramphenicol (Cm) (20 µg/mL), and spectinomycin (Spc) (50 µg/mL) were added as appropriate for plasmid-bearing cell growth and transformant selection. Bacterial growth was monitored with a Mini photo 518R (660 nm; TAITEC Co., Japan).

### Lysis assay

*E. coli* BL21 Δ*recA* (DE3) cells carrying a plasmid encoding one of the Sgl^PP7^ derivatives were grown overnight in LB medium supplemented with the appropriate antibiotics. The cultures were diluted 1:10,000 in phosphate-buffered saline and spotted onto LB agar plates supplemented with 0.2% (w/v) L-arabinose and the necessary antibiotics. The plates were incubated at 37 °C for 22 h and subsequently imaged.

### Sample preparation of the MurJ/Sgl^PP7^ complex

*E. coli* BL21 Δ*recA* (DE3) cells harboring the plasmids pKG537, pKH32, and pRM1210, encoding Met-Ala-*Ec*MurJ_2-511_ (from *E. coli* JCM 20135)-3× FLAG, Met-His_10_-Sgl^PP7^, and *Bs*Amj, respectively, were cultured in LB medium (Lennox, Nacalai) supplemented with 100 µg/mL Amp, 20 µg/mL Cm, and 50 µg/mL Spc at 37 °C. Protein expression was induced with 1 mM isopropyl β-D-thiogalactopyranoside (IPTG) and 0.2% (w/v) L-arabinose. Protein purification was performed as described previously [19]. The membrane fractions were isolated by ultracentrifugation and solubilized in buffer containing 2% n-dodecyl β-maltoside (DDM), 0.02% lauryl maltose neopentyl glycol (LMNG), and 0.002% cholesteryl hemisuccinate (CHS). The protein was subsequently purified using Ni-NTA agarose resin (QIAGEN), followed by a Superose 6 Increase 10/300 GL column (Cytiva). The MurJ/Sgl^PP7^ complex was finally purified in buffer (20 mM Tris-HCl (pH 8.0), 300 mM NaCl, 0.005% LMNG, 0.0005% CHS, and 0.1 mM PMSF) and concentrated to ~20 mg/mL using an Amicon Ultra 30 K NMWL (MERCK). Nanodisc reconstitution was performed as described previously [19]. The purified MurJ/Sgl^PP7^ complex was mixed with MSP1D1 [31] and *E. coli* phospholipids (Avanti) at a molar ratio of 1.4:1:120 in buffer (20 mM Tris-HCl (pH 8.0), 300 mM NaCl, 0.005% LMNG, 0.0005% CHS, and 0.1 mM PMSF). The mixture was incubated at 4 °C for 1 h. Then, the detergent was removed by Bio-Beads SM2 (Bio-Rad). The resulting nanodiscs were further purified using a Superdex 200 Increase 10/300 GL column preequilibrated with buffer (20 mM Tris-HCl (pH 8.0), 300 mM NaCl, and 0.1 mM PMSF). The MurJ/Sgl^PP7^-reconstituted nanodisc was concentrated using an Amicon Ultra 50 K NMWL.

### Cryo-EM data collection and processing

Quantifoil holey carbon grids (Cu R1.2/R1.3, 300 mesh) were glow-discharged at 7 Pa and 10 mA for 10 s using a JEC-3000FC sputter coater (JEOL) before sample application. A 3 μL aliquot of 2.8 mg/mL MurJ/Sgl^PP7^ complex-reconstituted nanodiscs was applied onto the grid, blotted for 2 s at 100% humidity, 8 °C, and blot force 10, and plunged into liquid ethane using a Vitrobot Mark IV (Thermo Fisher Scientific).

The cryo-EM dataset was collected on a CRYO ARM 300 transmission electron microscope (JEOL) operated at an accelerating 300 kV, equipped with a cold-field emission gun, an in-column Omega-type energy filter, and a Gatan K3 direct electron detector (Gatan) at SPring-8. Images were collected at a nominal magnification of 60,000×, corresponding to a calibrated pixel size of 0.752 Å/pixel, 50 frames per image with a total exposure dose of 50 e^−^/Å^2^ using a SerialEM [32].

Data processing was performed using cryoSPARC v4.6.2–v5.0.6 software [33]. The cryo-EM data-processing workflow for the MurJ/Sgl^PP7^ complex is shown in S1D Fig. A total of 17,650 movies were aligned using patch motion correction, and the contrast transfer function (CTF) parameters were estimated using the Patch CTF estimation. The particles were automatically picked using the Blob picker in cryoSPARC from 200 of the 17,650 micrographs. Particles were extracted with downsampling via Fourier cropping to a pixel size of 1.504 Å and subjected to 2D classification, generating 2D class averages. Using these 2D class averages as templates, particles were repicked using the Template Picker from 17,650 micrographs, yielding 7,554,685 particles. The repicked particles were extracted with downsampling via Fourier cropping to the pixel size of 1.504 Å and subjected to five rounds of 2D classification (*K* = 350, 350, 300, 250, 250). After 2D classification, 1,777,257 high-quality particles were subjected to ab initio 3D reconstruction (*K* = 4), generating initial 3D models. After two rounds of 2D classification, the 3,749,310 particles were then subjected to four rounds of heterogeneous refinement (*K* = 4, 4, 4, 4) using the initial models as references, removing junk particles. The best 3D class, containing 453,217 particles, was re-extracted to 0.752 Å/pixel and refined using Non-Uniform (NU) refinement. After NU refinement, these particles were subjected to reference-based motion correction and then to NU refinement, and local refinement using the mask covering the entire complex, including the nanodisc. These processes yielded a 3D cryo-EM map with an estimated overall resolution of 3.2 Å. A sharpened map was generated using the Sharpening Tool in cryoSPARC with a B-factor of −100 Å^2^. Local resolution was estimated using the Local Resolution Estimation job in cryoSPARC.

### Model building and refinement

The Sgl^PP7^ structure was predicted using AlphaFold3 [21]. The predicted Sgl^PP7^ model and an EcMurJ outward model from the MurJ/Lys^M^ complex structure (PDB ID: 9UKV) were docked into the cryo-EM map using UCSF ChimeraX. The model was manually modified in Coot [34], and further refinement was conducted using phenix.real_space_refine in PHENIX [35]. Data processing and refinement statistics are provided in S1 Fig and S1 Table. The final molecular model and cryo-EM map were visualized using PyMOL (https://pymol.org/) or UCSF ChimeraX [36].

### MD simulations of MurJ and the MurJ/Sgl^PP7^ complex in membrane

MD simulations were performed for two systems, the MurJ/Sgl^PP7^ complex and Sgl^PP7^-free MurJ using YASARA dynamics software [37] (https://www.yasara.org/). The simulations were set up using the membrane MD macro (md_runmembrane.mcr). Each system was embedded in a mixed palmitoyloleoylphosphatidylethanolamine/palmitoyloleoylphosphatidylglycerol (POPE/POPG) lipid bilayer and solvated in a 0.9% NaCl solution. The hydrogen-bonding network was optimized before simulation to improve structural stability [38]. After steepest-descent and simulated-annealing energy minimization to remove steric clashes, production simulations were carried out for 120 ns using the AMBER14 force field [39]. Van der Waals interactions were calculated with an 8 Å cutoff, whereas long-range electrostatic interactions were treated without a cutoff using the particle mesh Ewald algorithm [40,41]. The equations of motion were integrated using a multiple time-step scheme with time steps of 1.25 fs for bonded interactions and 2.5 fs for non-bonded interactions under NPT conditions at 298 K and 1 atm, as implemented in YASARA [42]. To reduce bias, three independent simulations were performed using distinct POPE/POPG membrane environments. For the MurJ/Sgl^PP7^ complex, Run 1 contained 169 POPE and 61 POPG lipids, Run 2 contained 175 POPE and 58 POPG lipids, and Run 3 contained 169 POPE and 64 POPG lipids. For Sgl^PP7^-free MurJ, Run 1 contained 177 POPE and 60 POPG lipids, Run 2 contained 173 POPE and 58 POPG lipids, and Run 3 contained 174 POPE and 64 POPG lipids. The Cα RMSD of MurJ in each simulation was examined to verify the stability and flexibility of the complexes.

## Supporting information

S1 Fig Cryo-EM data processing workflow for the MurJ/Sgl^PP7^ complex. **(A)** Size-exclusion chromatogram of the MurJ/Sgl^PP7^-reconstituted nanodisc containing *Ec*MurJ-3×FLAG, His_10_-Sgl^PP7^ and MSP1D1, run on a Superdex 200 Increase 10/300 GL column. The asterisked fraction was analyzed by SDS-PAGE and stained with Coomassie Brilliant Blue. **(B)** Representative electron micrograph of the nanodisc-reconstituted MurJ/Sgl^PP7^ complex. **(C)** 2D class averages of the MurJ/Sgl^PP7^ complex. **(D)** Image processing workflow for the MurJ/Sgl^PP7^ complex. The local resolution of the reconstructed map was estimated using cryoSPARC. **(E)** Gold-standard FSC curve used for global resolution estimation within cryoSPARC. **(F)** Cryo-EM map of Sgl^PP7^ and its model. The sharpened cryo-EM map around the modeled region of Sgl^PP7^ was displayed as a mesh at 4.4 σ using PyMOL. Sgl^PP7^ is shown in cartoon representation, and its side chains are shown as sticks. The underlying data for (A) can be found in S1 Data. The original gel images of (A) can be found in S1 Raw Images.
(PDF)

S2 Fig Comparison of MurJ/Sgl^PP7^ structures. **(A)** Schematic representations of the Sgl^PP7^ and MurJ constructs used in this structural study and the previously reported structural study of MurJ/Sgl^PP7^ complexes. **(B)** Sharpened cryo-EM maps of the MurJ/Sgl^PP7^ complexes from this study (left) and the previous study (right). Resolutions and contour levels are indicated in the figure. **(C)** Close-up views of the cryo-EM maps and atomic models around the N- and C-terminal regions of Sgl^PP7^ from this study (left) and the previous study (right). The sharpened maps are displayed in light blue at a contour level of 0.032 for this study and 0.07 for the previous study. The unsharpened map from this study is displayed in white at a contour level of 0.016 (left). The Sgl^PP7^ models from this study and the previous study are shown in green and magenta, respectively. Red asterisks indicate alanine substitutions in the construct used in the previous study. **(D)** Superposition of the ribbon models from this study and the previous study. MurJ is shown in gray.
(PDF)

S3 Fig AlphaFold-predicted models of MurJ/Sgl^PP7^ complex. MurJ/Sgl^PP7^ models were generated using AlphaFold3 [21] (https://alphafoldserver.com/). **(A)** Predicted models color-coded by per-residue pLDDT scores. **(B)** Predicted models coloured light blue (MurJ) and green (Sgl^PP7^).
(PDF)

S4 Fig Dispensability of the extreme N-terminal region of Sgl^PP7^. **(A)** Schematic representations of full-length Sgl^PP7^ and GFP-fused Sgl^PP7^ constructs used for N-terminal truncation analysis. **(B)** Lytic activity of N-terminally truncated Sgl^PP7^ mutants. *E. coli* cells harboring vector plasmids or plasmids encoding Sgl^PP7^ mutants were grown on agar plates with or without induction. Dark colonies indicate normal bacterial growth. The Sgl^PP7^ secondary structure and transmembrane region were predicted using the PSIPRED server (https://bioinf.cs.ucl.ac.uk/psipred/) and TMHMM v2.0 server (https://services.healthtech.dtu.dk/services/TMHMM-2.0/), respectively.
(PDF)

S5 Fig Uncropped spot assay images corresponding to Fig 2C. **(A)** Original images in Fig 2C (left) showing the cell lytic activities of the C-terminal-truncated His_10_-Sgl^PP7^ mutants. **(B)** Original images in Fig 2C (middle) showing the cell lytic activities of the His_10_-Sgl^PP7^ W53 substitution mutants. **(C)** Original images in Fig 2C (right) showing the cell lytic activities of the His_10_-Sgl^PP7^ (1–53) W53 substitution mutants.
(PDF)

S6 Fig Lipid-like map bound to the MurJ/Sgl^PP7^ complex. **(A)** Cryo-EM map and cartoon model of the MurJ/Sgl^PP7^ complex, highlighting an unmodeled lipid-like map (orange) in the MurJ groove. MurJ and Sgl^PP7^ are colored as in Fig 1F. **(B)** Close-up view of the lipid-like map. MurJ residues implicated in substrate binding, F22, R24, D25, and R270, are shown as sticks. (C) Conformation-dependent rearrangements of residues in the putative substrate-binding site of MurJ. Cartoon representations and close-up views of the TM1–TM8 region are shown for the inward-open, inward-occluded, and outward-facing conformations of *Thermosipho africanus* MurJ and the inward-closed and outward-facing conformations of *Escherichia coli* MurJ (PDB IDs are indicated). The N-lobe (TM1–6), C-lobe (TM7–12), and TM13–14 are colored light purple, cyan, and gray, respectively. Sgl^PP7^ is shown in green. R24, D25, and R255 in *T. africanus* MurJ and F22, R24, D25, and R270 in *E. coli* MurJ are shown as sticks. R255 in *T. africanus* MurJ corresponds to R270 in *E. coli* MurJ. Distances between R24 and R255/R270 are shown in red, whereas distances involving D25 are shown in black.
(PDF)

S7 Fig Comparison of MurJ/Sgl structures. **(A)** Cartoon models of outward-facing MurJ in complex with Sgl^PP7^, truncated Sgl^PP7^, truncated Sgl^CJ3^, Lys^M^_1–37_, and Lys^M^_1–30_. MurJ N-lobe, C-lobe, and TM13–14 are colored purple, cyan, and gray, respectively. Sgl^PP7^, Sgl^CJ3^, and Lys^M^ are shown in green, purple, and orange, respectively. BRIL is shown in blue. PDB IDs are indicated above each panel. **(B)** Superposition of the transmembrane regions of Sgl^PP7^ and Sgl^CJ3^ bound to MurJ. MurJ TM2 and TM7 are shown in gray, and Sgl^PP7^ and Sgl^CJ3^ are shown in green and purple, respectively. Side chains are shown as sticks. **(C)** Amino acid sequences of Sgl^PP7^, Sgl^CJ3^, and Lys^M^. Helical regions in comparable structural positions are highlighted in blue. The C-terminal embedded tails of Sgl^PP7^ and Sgl^CJ3^ are highlighted in red.
(PDF)

S8 Fig Structural comparison of the MurJ-binding modes of Sgl^PP7^, Lys^M^, and Sgl^CJ3^. **(A)** Close-up views of the interaction interfaces near TM2 and TM7 in the MurJ/Sgl^PP7^, MurJ/Lys^M^, and MurJ/Sgl^CJ3^ complexes. Cartoon models of MurJ and Sgl proteins are colored as in S7 Fig. The side chains of Y36 of Sgl^PP7^, L14 of Lys^M^, and Y76 of Sgl^CJ3^, which insert into a similar pocket of MurJ, are shown as sticks. The side chains of MurJ residues surrounding this pocket are also shown as sticks. The C-terminal regions of Sgl^PP7^ and Sgl^CJ3^ are rendered transparent for clarity. **(B)** Surface representations of MurJ showing the distinct binding modes of Sgl^PP7^, Lys^M^, and Sgl^CJ3^. MurJ is shown as an electrostatic potential surface, colored from red (negative) to blue (positive). Sgl proteins are displayed in cartoon representation. Cutaway views of MurJ are shown in the MurJ/Sgl^PP7^ and MurJ/Sgl^CJ3^ complexes to visualize the internal cavity in the lower panels. **(C)** Close-up views of the C-terminal regions of Sgl^PP7^, Lys^M^, and Sgl^CJ3^ surrounded by TM2, TM7, TM8, TM10, and TM12 of MurJ. Sgl residues implicated in lytic activity by functional analyses in this study and previous studies are shown as sticks and labeled. MurJ residues positioned within 4.5 Å of the C-terminal region of each Sgl are shown as sticks.
(PDF)

S9 Fig Alanine-scanning analysis of the functionally required region of Sgl^PP7^. Lytic activity of alanine-substitution mutants of full-length Sgl^PP7^ (1–55). *E. coli* cells harboring vector plasmids or plasmids encoding the indicated mutants were grown on agar plates with or without induction. Dark colonies indicate normal bacterial growth.
(PDF)

S1 Table Cryo-EM data collection and refinement statistics. (PDF)

S2 Table Plasmids and strains used in this study. (PDF)

S1 Raw Images Raw data for Figs 1E and S1A. (PDF)

S1 Data Underlying data. Raw data for Figs 1D, 3A, 3C, 4C, and S1A.
(XLSX)

## Abbreviations

CHS: cholesteryl hemisuccinate
CTF: contrast transfer function
LMNG: lauryl maltose neopentyl glycol
MD: molecular dynamics
NU: Non-Uniform
PDB: Protein Data Bank
RMSD: root mean square deviation
Sgl: single-gene lysis
