# Supplementary material for: Structural basis for inhibition of the lipid flippase MurJ by phage lysis protein SglPP7 suggests convergent evolution with other Sgl peptides
Source: PLoS Biol. 2026 Oct 27;24(10):e3004039. doi: 10.1371/journal.pbio.3004039 (PMC13712586; doi:10.1371/journal.pbio.3004039)
Supplement: Supplementary file 1 [file pbio.3004039.s001.pdf]

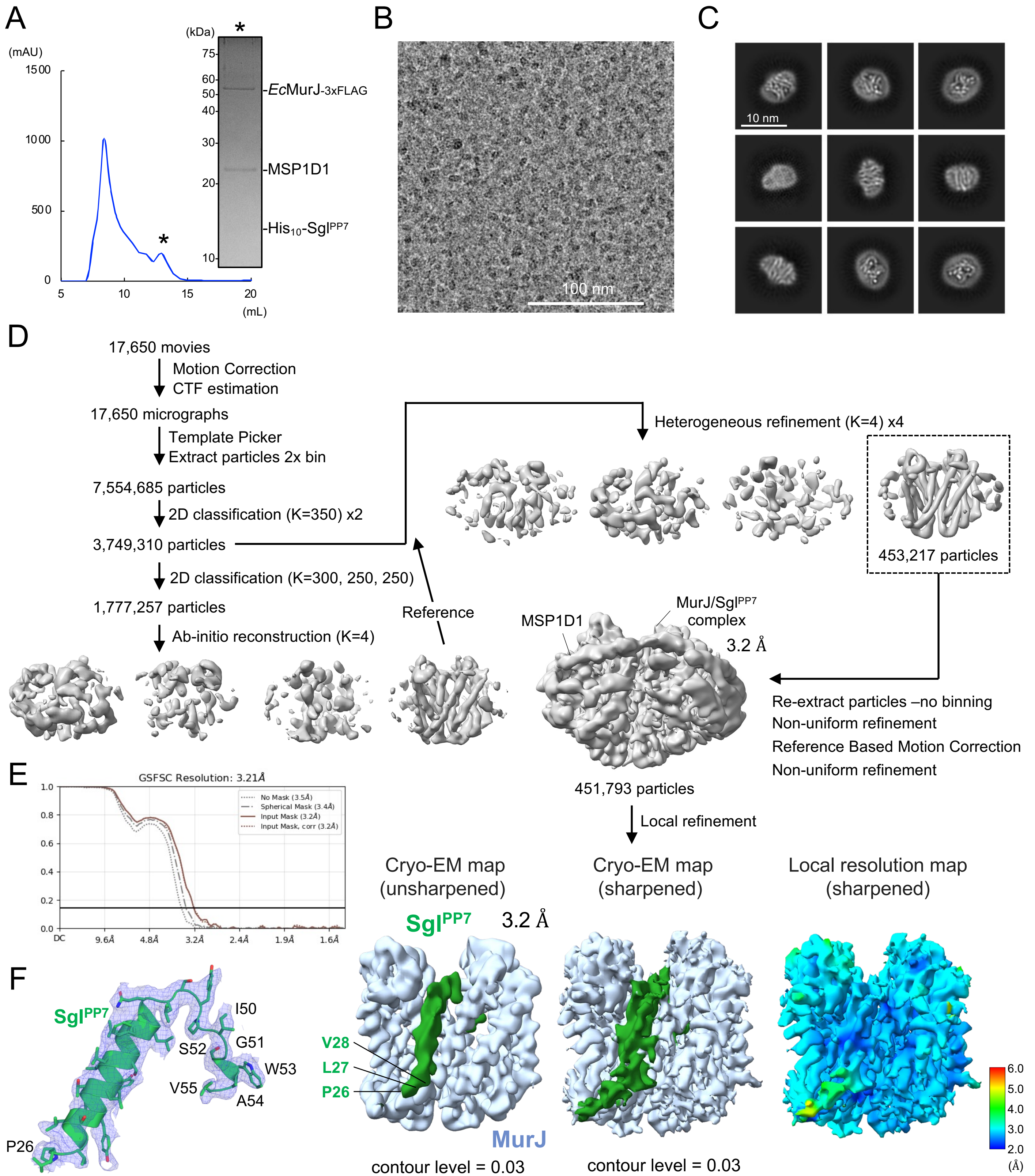
A
(mAU)
1500
1000
500
0
5
10
15
20
(mL)
*
(kDa)
75
60
50
40
30
20
10
-EcMurJ-3xFLAG
-MSP1D1
-His10-SglPP7
B
100 nm
C
10 nm
D
17,650 movies
Motion Correction
CTF estimation
17,650 micrographs
Template Picker
Extract particles 2x bin
7,554,685 particles
2D classification (K=350) x2
3,749,310 particles
2D classification (K=300, 250, 250)
1,777,257 particles
Ab-initio reconstruction (K=4)
Reference
Heterogeneous refinement (K=4) x4
453,217 particles
MSP1D1
MurJ/SglPP7 complex
3.2 Å
Re-extract particles –no binning
Non-uniform refinement
Reference Based Motion Correction
Non-uniform refinement
451,793 particles
Local refinement
E
GSFSC Resolution: 3.21Å
No Mask (3.5Å)
Spherical Mask (3.4Å)
Input Mask (3.2Å)
Input Mask, corr (3.2Å)
DC
9.6Å
4.8Å
3.2Å
2.4Å
1.9Å
1.6Å
Cryo-EM map (unsharpened)
Cryo-EM map (sharpened)
Local resolution map (sharpened)
SglPP7
3.2 Å
V28
L27
P26
MurJ
contour level = 0.03
contour level = 0.03
6.0
5.0
4.0
3.0
2.0
(Å)
F
SglPP7
I50
G51
S52
W53
V55
A54
P26
