# Supplementary material for: Structural basis for inhibition of the lipid flippase MurJ by phage lysis protein SglPP7 suggests convergent evolution with other Sgl peptides
Source: PLoS Biol. 2026 Oct 27;24(10):e3004039. doi: 10.1371/journal.pbio.3004039 (PMC13712586; doi:10.1371/journal.pbio.3004039)
Supplement: Supplementary file 2 [file pbio.3004039.s002.pdf]

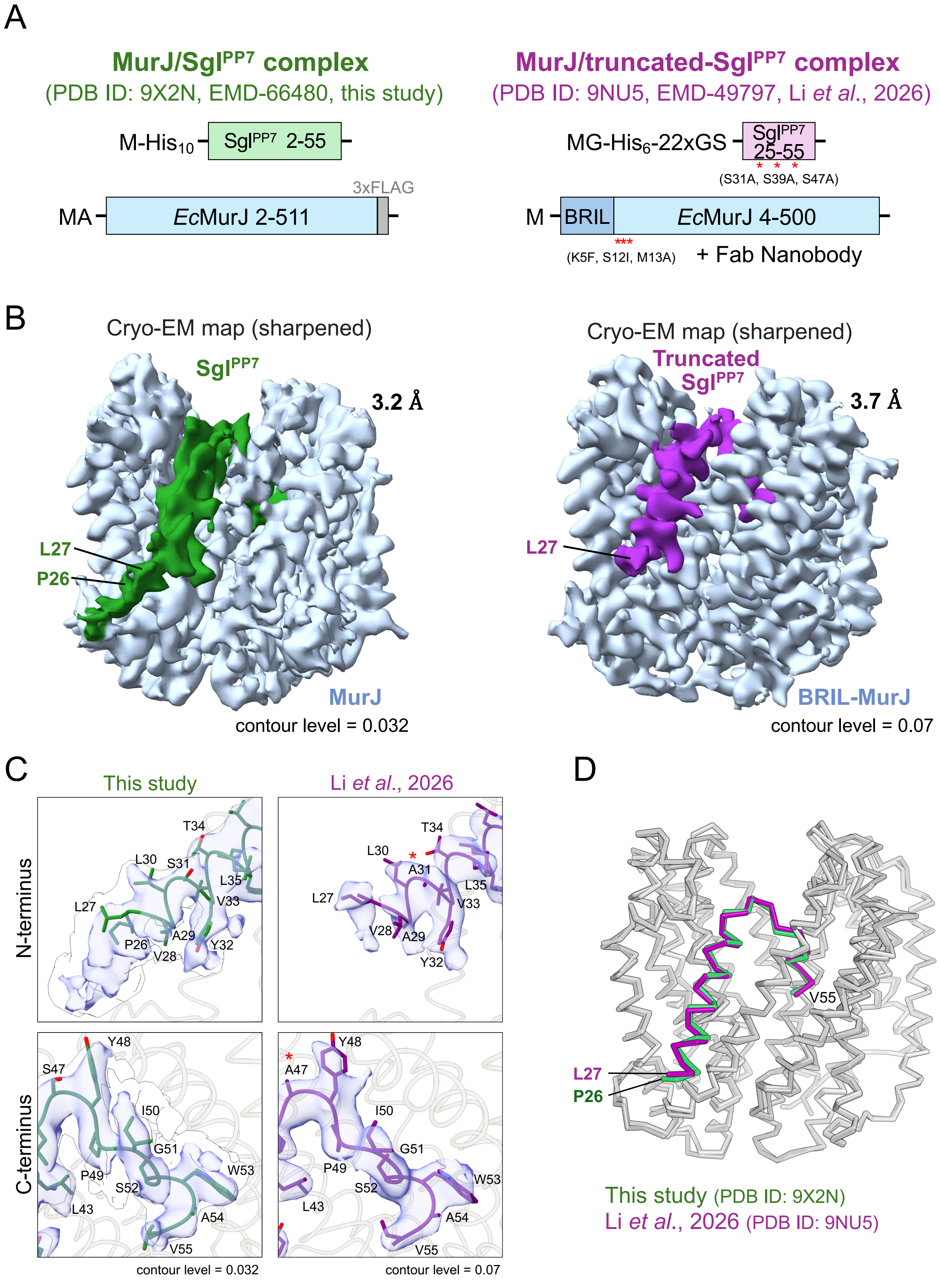
A
MurJ/Sgl^PP7 complex
(PDB ID: 9X2N, EMD-66480, this study)
M-His10
Sgl^PP7 2-55
3xFLAG
MA
EcMurJ 2-511
MurJ/truncated-Sgl^PP7 complex
(PDB ID: 9NU5, EMD-49797, Li et al., 2026)
MG-His6-22xGS
Sgl^PP7 25-55
(S31A, S39A, S47A)
M
BRIL
EcMurJ 4-500
(K5F, S12I, M13A)
+ Fab Nanobody
B
Cryo-EM map (sharpened)
Sgl^PP7
3.2 Å
L27
P26
MurJ
contour level = 0.032
Cryo-EM map (sharpened)
Truncated Sgl^PP7
3.7 Å
L27
BRIL-MurJ
contour level = 0.07
C
This study
Li et al., 2026
N-terminus
C-terminus
contour level = 0.032
contour level = 0.07
D
V55
L27
P26
This study (PDB ID: 9X2N)
Li et al., 2026 (PDB ID: 9NU5)
