# Supplementary material for: Structural basis for inhibition of the lipid flippase MurJ by phage lysis protein SglPP7 suggests convergent evolution with other Sgl peptides
Source: PLoS Biol. 2026 Oct 27;24(10):e3004039. doi: 10.1371/journal.pbio.3004039 (PMC13712586; doi:10.1371/journal.pbio.3004039)
Supplement: Supplementary file 3 [file pbio.3004039.s003.pdf]

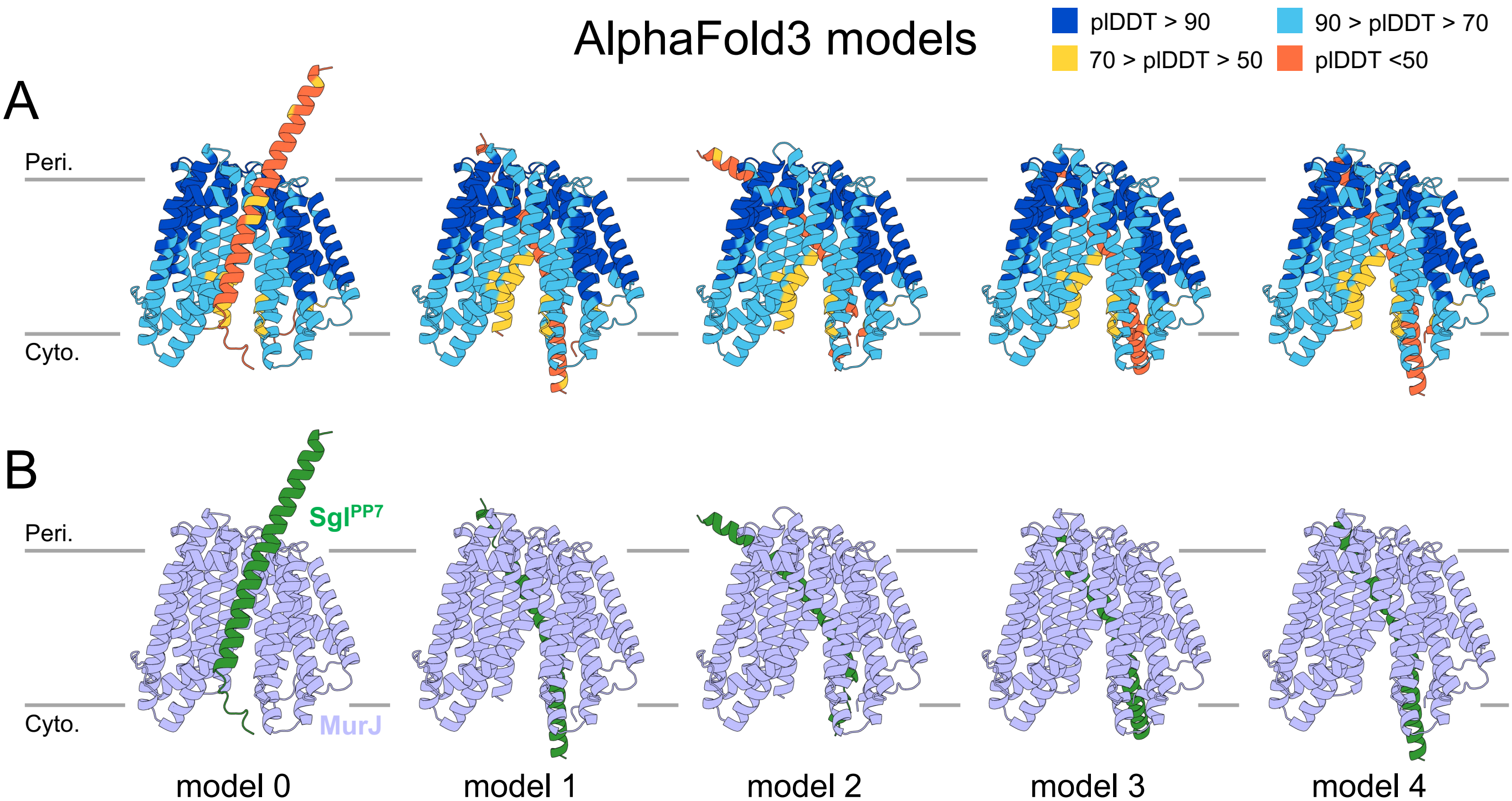

AlphaFold3 models
pIDDT > 90
90 > pIDDT > 70
70 > pIDDT > 50
pIDDT <50
A
Peri.
Cyto.
B
Peri.
Cyto.
Sgl^PP7
MurJ
model 0
model 1
model 2
model 3
model 4
