# Supplementary material for: Structural basis for inhibition of the lipid flippase MurJ by phage lysis protein SglPP7 suggests convergent evolution with other Sgl peptides
Source: PLoS Biol. 2026 Oct 27;24(10):e3004039. doi: 10.1371/journal.pbio.3004039 (PMC13712586; doi:10.1371/journal.pbio.3004039)
Supplement: Supplementary file 4 [file pbio.3004039.s004.pdf]

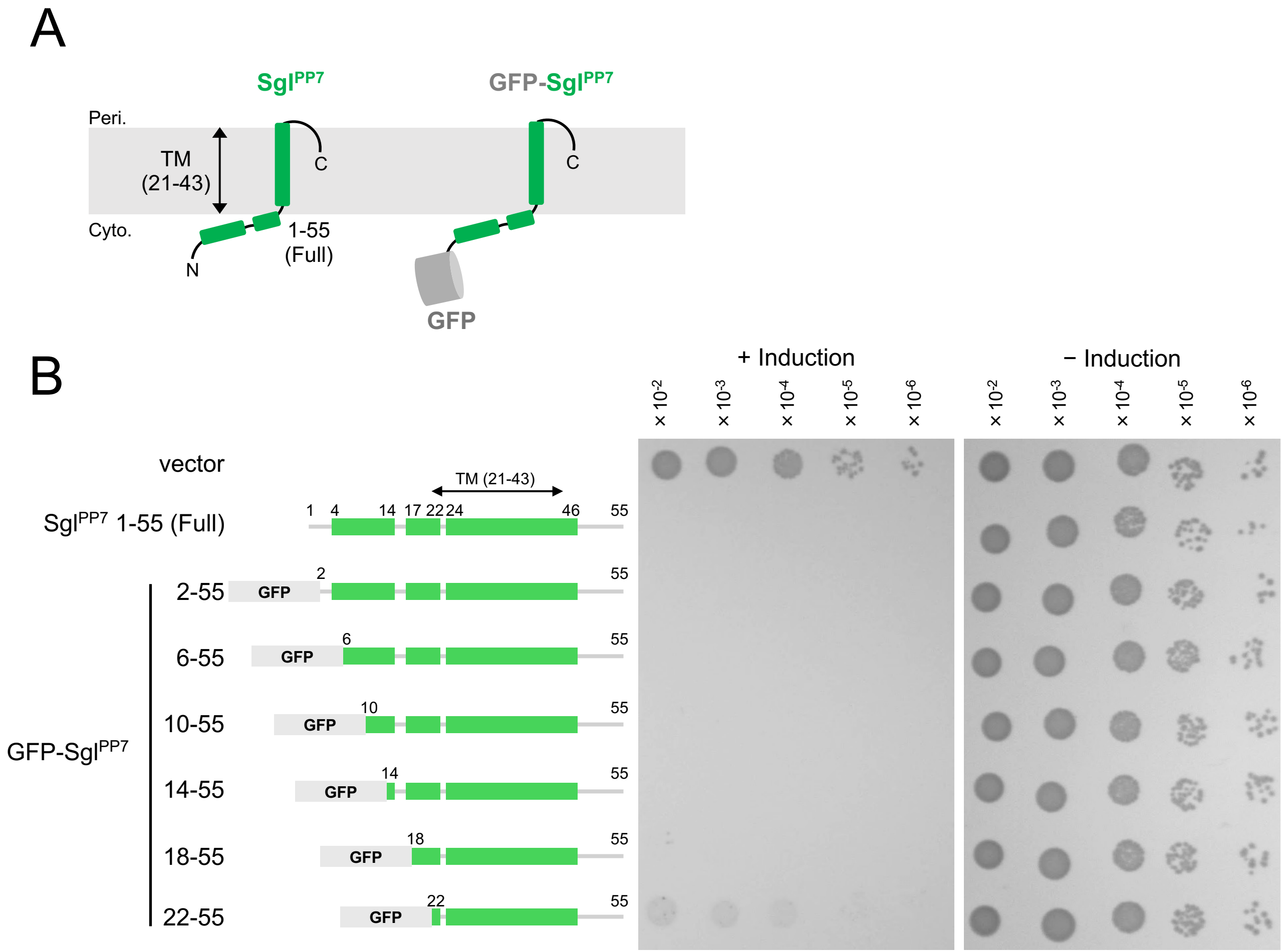
A
Sgl^{PP7}
GFP-Sgl^{PP7}
Peri.
TM (21-43)
C
Cyto.
1-55 (Full)
N
GFP
B
+ Induction
− Induction
× 10-2
× 10-3
× 10-4
× 10-5
× 10-6
vector
Sgl^{PP7} 1-55 (Full)
TM (21-43)
1 4 14 17 22 24 46 55
GFP-Sgl^{PP7}
2-55
6-55
10-55
14-55
18-55
22-55
GFP
