# Supplementary material for: Structural basis for inhibition of the lipid flippase MurJ by phage lysis protein SglPP7 suggests convergent evolution with other Sgl peptides
Source: PLoS Biol. 2026 Oct 27;24(10):e3004039. doi: 10.1371/journal.pbio.3004039 (PMC13712586; doi:10.1371/journal.pbio.3004039)
Supplement: Supplementary file 5 [file pbio.3004039.s005.pdf]

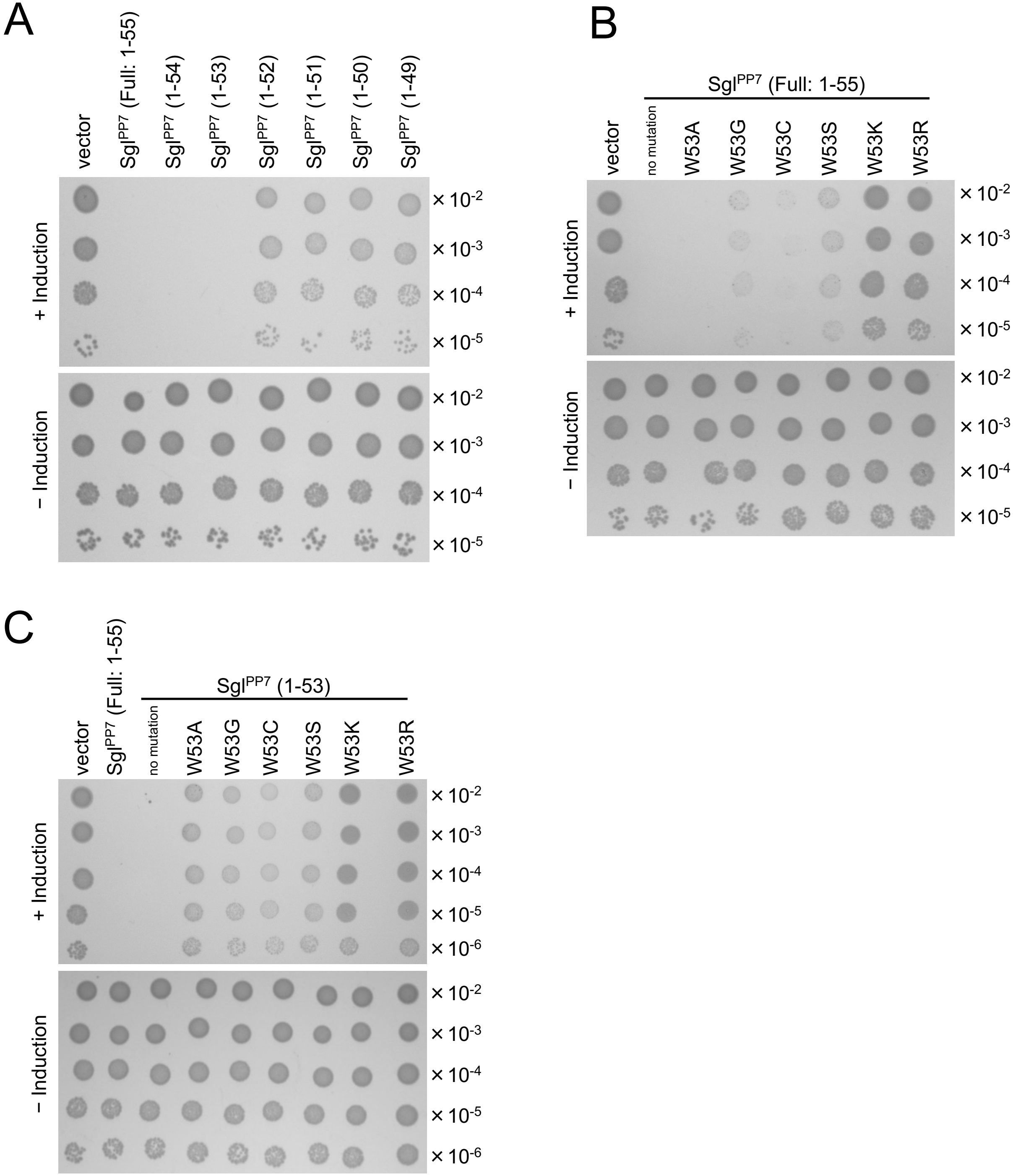
A
vector
SglPP7 (Full: 1-55)
SglPP7 (1-54)
SglPP7 (1-53)
SglPP7 (1-52)
SglPP7 (1-51)
SglPP7 (1-50)
SglPP7 (1-49)
+ Induction
− Induction
× 10-2
× 10-3
× 10-4
× 10-5
B
SglPP7 (Full: 1-55)
vector
no mutation
W53A
W53G
W53C
W53S
W53K
W53R
C
SglPP7 (1-53)
× 10-6
