# Supplementary material for: Structural basis for inhibition of the lipid flippase MurJ by phage lysis protein SglPP7 suggests convergent evolution with other Sgl peptides
Source: PLoS Biol. 2026 Oct 27;24(10):e3004039. doi: 10.1371/journal.pbio.3004039 (PMC13712586; doi:10.1371/journal.pbio.3004039)
Supplement: Supplementary file 6 [file pbio.3004039.s006.pdf]

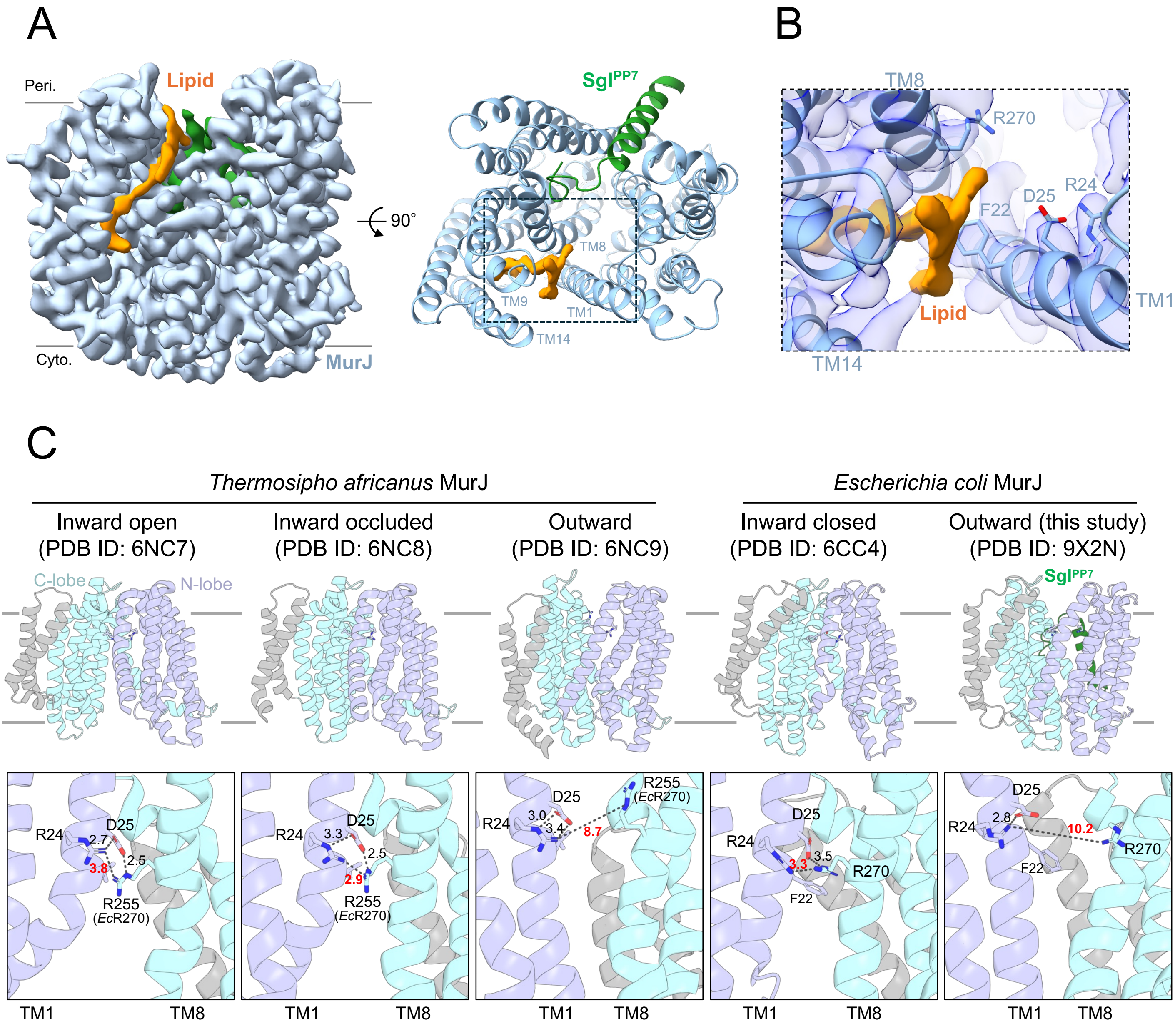

A
Peri.
Lipid
Cyto.
MurJ
90°
Sgl^PP7
TM8
TM9
TM1
TM14
B
TM8
R270
D25
R24
F22
Lipid
TM1
TM14
C
Thermosipho africanus MurJ
Escherichia coli MurJ
Inward open (PDB ID: 6NC7)
Inward occluded (PDB ID: 6NC8)
Outward (PDB ID: 6NC9)
Inward closed (PDB ID: 6CC4)
Outward (this study) (PDB ID: 9X2N)
C-lobe
N-lobe
Sgl^PP7
R24
D25
2.7
3.8
2.5
R255 (EcR270)
3.3
2.9
3.0
3.4
8.7
3.5
R270
F22
2.8
10.2
TM1
TM8
