# Supplementary material for: Structural basis for inhibition of the lipid flippase MurJ by phage lysis protein SglPP7 suggests convergent evolution with other Sgl peptides
Source: PLoS Biol. 2026 Oct 27;24(10):e3004039. doi: 10.1371/journal.pbio.3004039 (PMC13712586; doi:10.1371/journal.pbio.3004039)
Supplement: Supplementary file 7 [file pbio.3004039.s007.pdf]

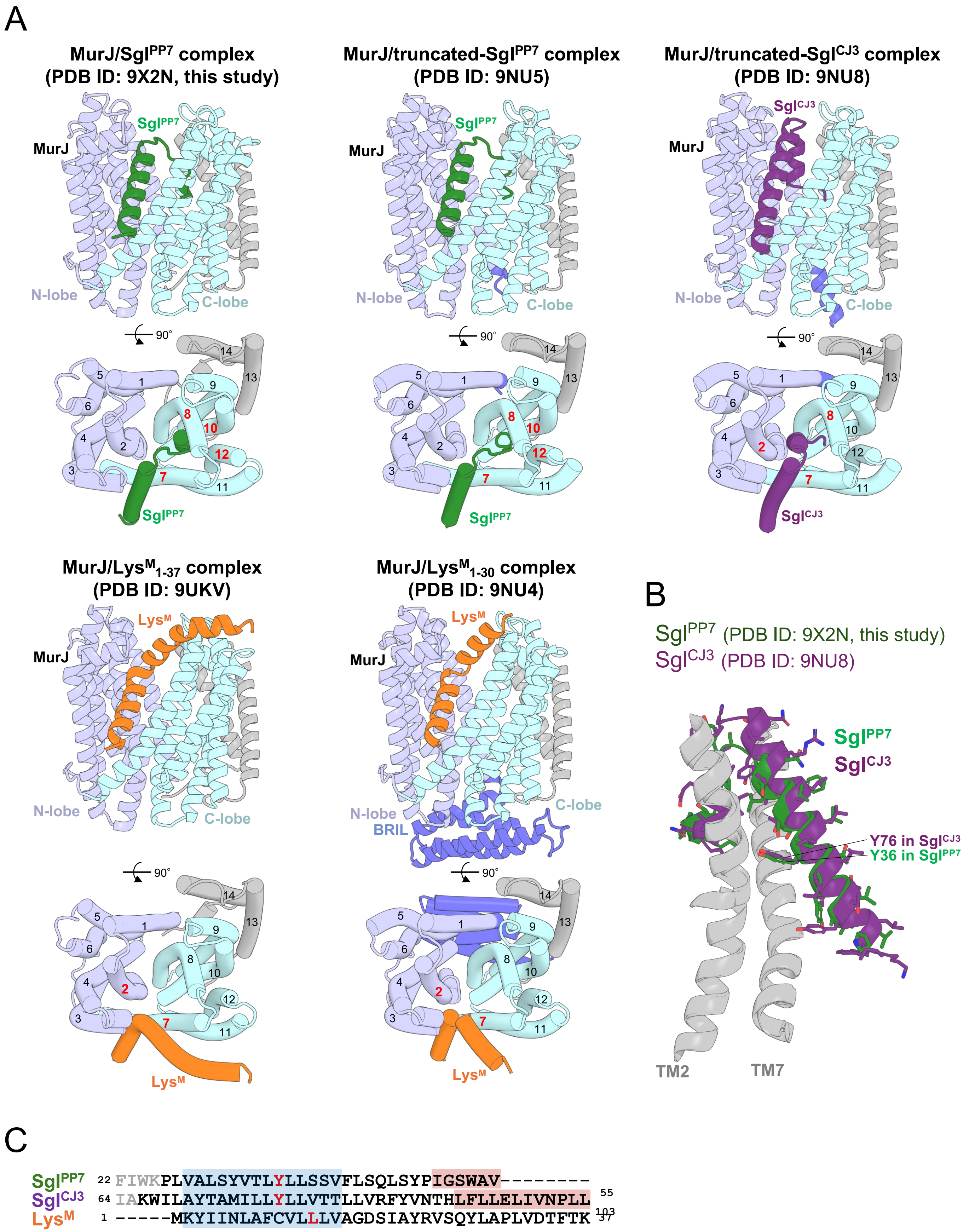

A
MurJ/Sgl^PP7 complex (PDB ID: 9X2N, this study)
MurJ/truncated-Sgl^PP7 complex (PDB ID: 9NU5)
MurJ/truncated-Sgl^CJ3 complex (PDB ID: 9NU8)
MurJ
Sgl^PP7
Sgl^CJ3
N-lobe
C-lobe
90°
MurJ/Lys^M 1-37 complex (PDB ID: 9UKV)
MurJ/Lys^M 1-30 complex (PDB ID: 9NU4)
Lys^M
BRIL
B
Sgl^PP7 (PDB ID: 9X2N, this study)
Sgl^CJ3 (PDB ID: 9NU8)
Y76 in Sgl^CJ3
Y36 in Sgl^PP7
TM2
TM7
C
Sgl^PP7 22 FIWKPLVALSYVTLYLLSSVFLSQLSYPIGSWAV-------- 55
Sgl^CJ3 64 IAKWILAYTAMILLYLLVTTLLVRFYVNTHLFLLELIVNPLL 103
Lys^M 1 -----MKYIINLAFCVLLLVAGDSIAYRVSQYLAPLVDTFTK 37
