# Supplementary material for: Structural basis for inhibition of the lipid flippase MurJ by phage lysis protein SglPP7 suggests convergent evolution with other Sgl peptides
Source: PLoS Biol. 2026 Oct 27;24(10):e3004039. doi: 10.1371/journal.pbio.3004039 (PMC13712586; doi:10.1371/journal.pbio.3004039)
Supplement: Supplementary file 8 [file pbio.3004039.s008.pdf]

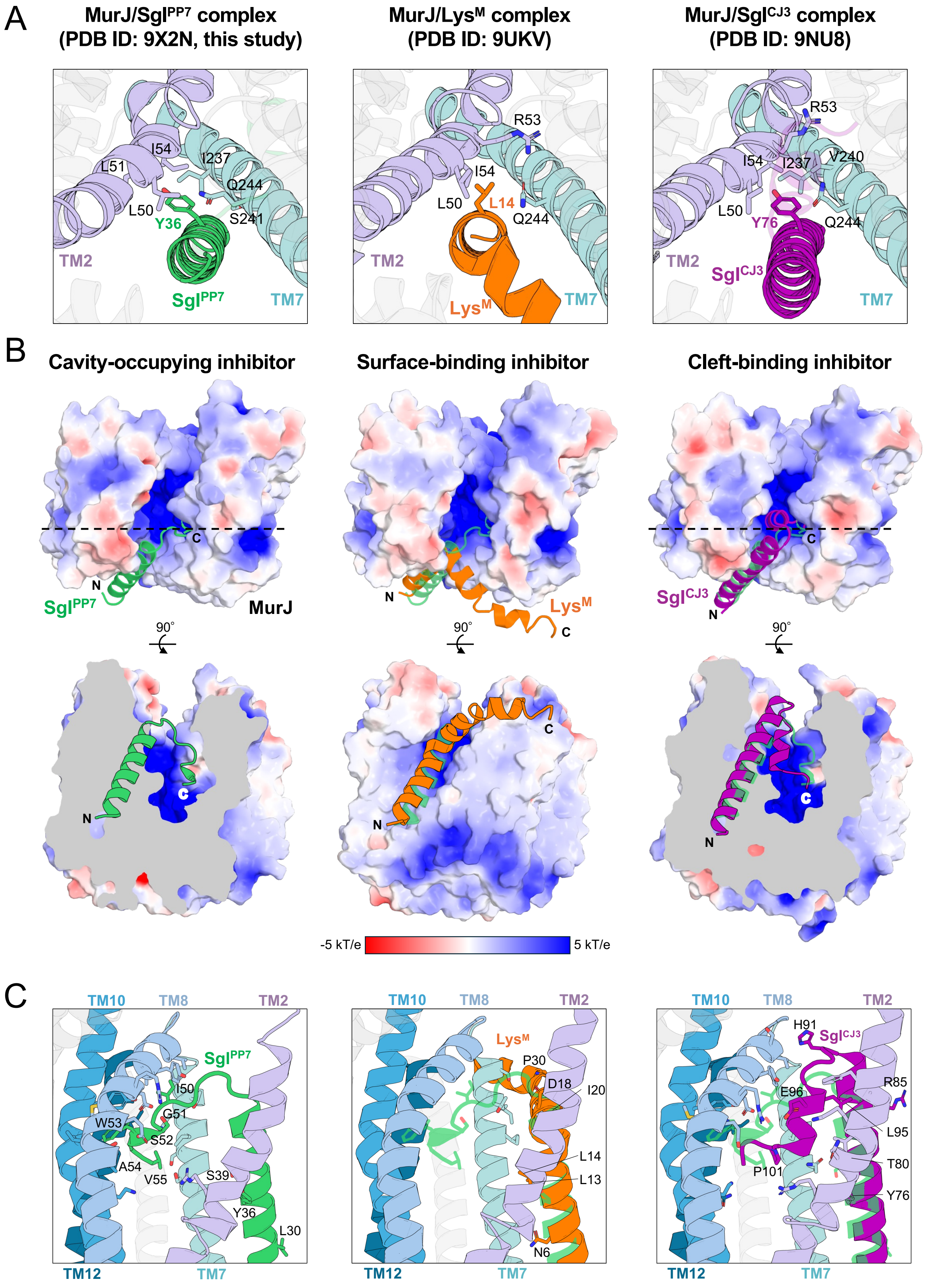

A
MurJ/Sgl^PP7 complex (PDB ID: 9X2N, this study)
MurJ/Lys^M complex (PDB ID: 9UKV)
MurJ/Sgl^CJ3 complex (PDB ID: 9NU8)
L51
I54
I237
Q244
L50
Y36
S241
TM2
Sgl^PP7
TM7
R53
I54
L50
L14
Q244
TM2
Lys^M
TM7
R53
I54
I237
V240
L50
Y76
Q244
TM2
Sgl^CJ3
TM7
B
Cavity-occupying inhibitor
Surface-binding inhibitor
Cleft-binding inhibitor
C
N
Sgl^PP7
MurJ
N
Lys^M
C
C
Sgl^CJ3
N
90°
90°
90°
N
C
C
N
C
N
-5 kT/e
5 kT/e
C
TM10
TM8
TM2
Sgl^PP7
I50
G51
W53
S52
A54
V55
S39
Y36
L30
TM12
TM7
TM10
TM8
TM2
Lys^M
P30
D18
I20
L14
L13
N6
TM12
TM7
TM10
TM8
TM2
H91
Sgl^CJ3
R85
E96
L95
T80
P101
Y76
TM12
TM7
