# Supplementary material for: Structural basis for inhibition of the lipid flippase MurJ by phage lysis protein SglPP7 suggests convergent evolution with other Sgl peptides
Source: PLoS Biol. 2026 Oct 27;24(10):e3004039. doi: 10.1371/journal.pbio.3004039 (PMC13712586; doi:10.1371/journal.pbio.3004039)
Supplement: Supplementary file 9 [file pbio.3004039.s009.pdf]

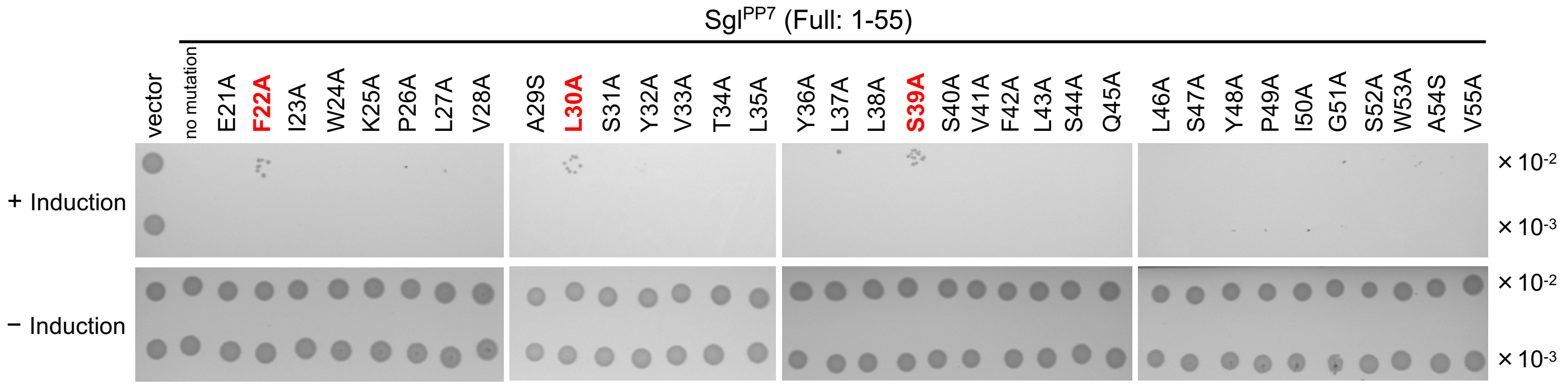

Sgl^{PP7} (Full: 1-55)
vector
no mutation
E21A
F22A
I23A
W24A
K25A
P26A
L27A
V28A
A29S
L30A
S31A
Y32A
V33A
T34A
L35A
Y36A
L37A
L38A
S39A
S40A
V41A
F42A
L43A
S44A
Q45A
L46A
S47A
Y48A
P49A
I50A
G51A
S52A
W53A
A54S
V55A
+ Induction
− Induction
× 10^{-2}
× 10^{-3}
× 10^{-2}
× 10^{-3}
