# Supplementary material for: Structural basis for inhibition of the lipid flippase MurJ by phage lysis protein SglPP7 suggests convergent evolution with other Sgl peptides
Source: PLoS Biol. 2026 Oct 27;24(10):e3004039. doi: 10.1371/journal.pbio.3004039 (PMC13712586; doi:10.1371/journal.pbio.3004039)
Supplement: Supplementary file 10 [file pbio.3004039.s010.pdf]

**Table S1. Data collection and refinement statistics.**

| | ***Ec*MurJ/Sgl$^{PP7}$-ND (EMD-66480, PDB ID: 9X2N)** |
|---|---|
| **Data collection and processing** | |
| Magnification | x60,000 |
| Voltage (kV) | 300 |
| Electron exposure (e–/Å$^2$) | 50 |
| Defocus range (μm) | -1.4 to -1.6 |
| Pixel size (Å) | 0.752 |
| Symmetry imposed | C1 |
| Initial particle images (no.) | 7,554,685 |
| Final particle images (no.) | 451,793 |
| Map resolution (Å) | 3.2 |
| FSC threshold | 0.143 |
| **Refinement** | |
| Initial model used (PDB code) | 9UKV and AlphaFold model |
| Model composition | |
| Non-hydrogen atoms | 4128 |
| Protein residues | 541 |
| Ligands | 0 |
| *B* factors (Å$^2$) | |
| Protein | 63.21 |
| Ligand | 0 |
| R.m.s. deviations | |
| Bond lengths (Å) | 0.005 |
| Bond angles (°) | 0.780 |
| **Validation** | |
| MolProbity score | 1.71 |
| Clashscore | 9.76 |
| Poor rotamer (%) | 0.23 |
| Ramachandran plot | |
| Favored (%) | 96.83 |
| Allowed (%) | 3.17 |
| Disallowed (%) | 0 |
