# Supplementary material for: Structural basis for inhibition of the lipid flippase MurJ by phage lysis protein SglPP7 suggests convergent evolution with other Sgl peptides
Source: PLoS Biol. 2026 Oct 27;24(10):e3004039. doi: 10.1371/journal.pbio.3004039 (PMC13712586; doi:10.1371/journal.pbio.3004039)
Supplement: Supplementary file 11 [file pbio.3004039.s011.pdf]

**Table S2. Plasmids and Strains used in this study.**

| Plasmids | Vector | Encoded gene and description | Reference or source |
|---|---|---|---|
| pBAD33 | - | Expression vector, $P_{araBAD}$, $Cm^R$ | *(30)* |
| pKK568 | pBAD33 | *$lys^M$* | *(17)* |
| pKG265 | pBAD33 | *$lys^M$-$his_{10}$* | This study |
| pKG360 | pBAD33 | *$his_{10}$-$lys^M$* | This study |
| pKG382 | pBAD33 | *$sgl^{PP7}$* | This study |
| pKG398 | pBAD33 | *$sgl^{PP7}$-$his_{10}$* | This study |
| pKG404 | pBAD33 | *$his_{10}$-$sgl^{PP7}$* | This study |
| pKG517 | pBAD33 | *gfp-$sgl^{PP7}$* (2-55) | This study |
| pKG751 | pBAD33 | *gfp-$sgl^{PP7}$* (6-55) | This study |
| pKG753 | pBAD33 | *gfp-$sgl^{PP7}$* (10-55) | This study |
| pKG755 | pBAD33 | *gfp-$sgl^{PP7}$* (14-55) | This study |
| pKG757 | pBAD33 | *gfp-$sgl^{PP7}$* (18-55) | This study |
| pKG759 | pBAD33 | *gfp-$sgl^{PP7}$* (22-55) | This study |
| pBAD18 | - | Expression vector, $P_{araBAD}$, $Amp^R$ | *(30)* |
| pKH32 | pBAD18 | *$his_{10}$-$sgl^{PP7}$* (1-55), Full-length | This study |
| pKH57 | pBAD18 | *$his_{10}$-$sgl^{PP7}$* (1-54) | This study |
| pKH40 | pBAD18 | *$his_{10}$-$sgl^{PP7}$* (1-53) | This study |
| pKH42 | pBAD18 | *$his_{10}$-$sgl^{PP7}$* (1-52) | This study |
| pKH59 | pBAD18 | *$his_{10}$-$sgl^{PP7}$* (1-51) | This study |
| pKH45 | pBAD18 | *$his_{10}$-$sgl^{PP7}$* (1-50) | This study |
| pKH48 | pBAD18 | *$his_{10}$-$sgl^{PP7}$* (1-49) | This study |
| pKG743 | pBAD18 | *$his_{10}$-$sgl^{PP7}$* (W53A) | This study |
| pKH69 | pBAD18 | *$his_{10}$-$sgl^{PP7}$* (W53G) | This study |
| pKH72 | pBAD18 | *$his_{10}$-$sgl^{PP7}$* (W53C) | This study |
| pKH75 | pBAD18 | *$his_{10}$-$sgl^{PP7}$* (W53S) | This study |
| pKH70 | pBAD18 | *$his_{10}$-$sgl^{PP7}$* (W53K) | This study |
| pKH68 | pBAD18 | *$his_{10}$-$sgl^{PP7}$* (W53R) | This study |
| pKH81 | pBAD18 | *$his_{10}$-$sgl^{PP7}$* (1-53, W53A) | This study |
| pKH77 | pBAD18 | *$his_{10}$-$sgl^{PP7}$* (1-53, W53G) | This study |
| pKH78 | pBAD18 | *$his_{10}$-$sgl^{PP7}$* (1-53, W53C) | This study |
| pKH79 | pBAD18 | *$his_{10}$-$sgl^{PP7}$* (1-53, W53S) | This study |
| pKH80 | pBAD18 | *$his_{10}$-$sgl^{PP7}$* (1-53, W53K) | This study |
| pKH76 | pBAD18 | *$his_{10}$-$sgl^{PP7}$* (1-53, W53R) | This study |
| pKG711 | pBAD18 | *$his_{10}$-$sgl^{PP7}$* (E21A) | This study |
| pKG712 | pBAD18 | *$his_{10}$-$sgl^{PP7}$* (F22A) | This study |

| | | | |
|---|---|---|---|
| pKG713 | pBAD18 | *his*$_{10}$-*sgl*$^{PP7}$ (I23A) | This study |
| pKG714 | pBAD18 | *his*$_{10}$-*sgl*$^{PP7}$ (W24A) | This study |
| pKG715 | pBAD18 | *his*$_{10}$-*sgl*$^{PP7}$ (K25A) | This study |
| pKG716 | pBAD18 | *his*$_{10}$-*sgl*$^{PP7}$ (P26A) | This study |
| pKG717 | pBAD18 | *his*$_{10}$-*sgl*$^{PP7}$ (L27A) | This study |
| pKG718 | pBAD18 | *his*$_{10}$-*sgl*$^{PP7}$ (V28A) | This study |
| pKG719 | pBAD18 | *his*$_{10}$-*sgl*$^{PP7}$ (A29S) | This study |
| pKH66 | pBAD18 | *his*$_{10}$-*sgl*$^{PP7}$ (L30A) | This study |
| pKG721 | pBAD18 | *his*$_{10}$-*sgl*$^{PP7}$ (S31A) | This study |
| pKG722 | pBAD18 | *his*$_{10}$-*sgl*$^{PP7}$ (Y32A) | This study |
| pKG723 | pBAD18 | *his*$_{10}$-*sgl*$^{PP7}$ (V33A) | This study |
| pKG724 | pBAD18 | *his*$_{10}$-*sgl*$^{PP7}$ (T34A) | This study |
| pKG725 | pBAD18 | *his*$_{10}$-*sgl*$^{PP7}$ (L35A) | This study |
| pKG726 | pBAD18 | *his*$_{10}$-*sgl*$^{PP7}$ (Y36A) | This study |
| pKG727 | pBAD18 | *his*$_{10}$-*sgl*$^{PP7}$ (L37A) | This study |
| pKG728 | pBAD18 | *his*$_{10}$-*sgl*$^{PP7}$ (L38A) | This study |
| pKG729 | pBAD18 | *his*$_{10}$-*gl*$^{PP7}$ (S39A) | This study |
| pKG730 | pBAD18 | *his*$_{10}$-*sgl*$^{PP7}$ (S40A) | This study |
| pKG731 | pBAD18 | *his*$_{10}$-*sgl*$^{PP7}$ (V41A) | This study |
| pKG732 | pBAD18 | *his*$_{10}$-*sgl*$^{PP7}$ (F42A) | This study |
| pKG733 | pBAD18 | *his*$_{10}$-*sgl*$^{PP7}$ (L43A) | This study |
| pKG734 | pBAD18 | *his*$_{10}$-*sgl*$^{PP7}$ (S44A) | This study |
| pKG735 | pBAD18 | *his*$_{10}$-*sgl*$^{PP7}$ (Q45A) | This study |
| pKG736 | pBAD18 | *his*$_{10}$-*sgl*$^{PP7}$ (L46A) | This study |
| pKG737 | pBAD18 | *his*$_{10}$-*sgl*$^{PP7}$ (S47A) | This study |
| pKG738 | pBAD18 | *his*$_{10}$-*sgl*$^{PP7}$ (Y48A) | This study |
| pKG739 | pBAD18 | *his*$_{10}$-*sgl*$^{PP7}$ (P49A) | This study |
| pKG740 | pBAD18 | *his*$_{10}$-*sgl*$^{PP7}$ (I50A) | This study |
| pKG741 | pBAD18 | *his*$_{10}$-*sgl*$^{PP7}$ (G51A) | This study |
| pKG742 | pBAD18 | *his*$_{10}$-*sgl*$^{PP7}$ (S52A) | This study |
| pKG743 | pBAD18 | *his*$_{10}$-*sgl*$^{PP7}$ (W53A) | This study |
| pKG744 | pBAD18 | *his*$_{10}$-*sgl*$^{PP7}$ (A54S) | This study |
| pKG745 | pBAD18 | *his*$_{10}$-*sgl*$^{PP7}$ (V55A) | This study |
| | | | |
| pSTV28 | - | Expression vector, $P_{lac}$, $Cm^R$ | Takara Bio |
| pKG537 | pSTV28 | *EcmurJ-3xflag* | *(17)* |
| pKG50 | pTSP1 | *EcmurJ* | This study |

| | | | |
|---|---|---|---|
| pKG59 | pTSP1 | *EcmurJ* (V229G) | This study |
| pKG678 | pTSP1 | *EcmurJ* (Q244P) | This study |
| pKG681 | pTSP1 | *EcmurJ* (I248S) | This study |
| pKG646 | pTSP1 | *EcmurJ* (I252F) | This study |
| pRM1210 | pHM1550 | *Bacillus subtilis amj* | *(17)* |

| Strains | Genotype | Reference or source |
|---|---|---|
| JM109 | *recA1*, *endA1*, *gyrA96*, *thi-1*, *hsdR17*(*rK*- *mK*+), *e14*- (*mcrA*-), *supE44*, *relA1*, Δ(*lac-proAB*)/F'〔*traD36*, *proAB*+, *lac Iq*, *lacZ*ΔM15〕 | Takara Bio |
| BL21 Δ*recA* (DE3) | *F-, ompT, hsdSB*（*rB-mB-*）*, gal, dcm,* Δ*recA,* Δ*endA* | Nippon gene |
