# Supplementary material for: Structural basis for inhibition of the lipid flippase MurJ by phage lysis protein SglPP7 suggests convergent evolution with other Sgl peptides
Source: PLoS Biol. 2026 Oct 27;24(10):e3004039. doi: 10.1371/journal.pbio.3004039 (PMC13712586; doi:10.1371/journal.pbio.3004039)
Supplement: Supplementary file 13 [file pbio.3004039.s016.pdf]

Response to the Reviewers

Reviewer #1:

Hosoda et al. present the cryo-EM structure of the complex between the essential E. coli lipid II flippase MurJ and a phage lysis protein, SglPP7. The authors provide structural, extensive mutational, and molecular dynamics (MD) simulation analyses between MurJ and SglPP7, revealing that SglPP7 binds along the intramembrane cleft of MurJ and inserts its C-terminal tail into the internal cavity, locking MurJ in an outward-facing conformation. Of particular interest is the comparison of SglPP7 with LysM (SglM), a previously characterized MurJ-targeting lysis protein. The study shows that SglPP7 and LysM employ distinct strategies to inhibit MurJ. These findings contribute to the advancement of our understanding of the evolution of unrelated phages, leading to the convergent evolution of distinct strategies to inhibit this essential host protein. The present study is comprehensive and represents an important contribution to the field, offering high-resolution insights into the elusive interactional environment inside the intramembrane. Nevertheless, the following recommendations are hereby made for the improvement of the manuscript.

We thank the reviewer for the positive assessment of our work and for the constructive comments. We have carefully addressed each point and revised the manuscript accordingly.

Major comments:

1. Lines 115-125: In the "A lipid-like molecule between TM1, TM8, and TM14 of MurJ" chapter, the authors primarily relied on analogical reasoning, interpreting an unassigned density inside the MurJ-SglPP7 complex as potentially mimicking lipid II binding. In their structure, the lipid-like feature is located near residues F22, R24, and R270. Furthermore, previous biochemical and structural studies have shown that R24, D25, and R270 are essential for cell viability and have been proposed to form the binding site for the phosphate group of lipid II. Although the similarity between these regions supports the authors' reasoning, notable differences remain in the binding sites; for protein-ligand interactions, even minor discrepancies can induce significant functional variations. Consequently, I remain cautious regarding the authors' direct interpretation of this density as a lipid II analog. I recommend that the authors provide a more detailed description of the specific roles and binding characteristics of R24, D25, and R270 as reported in published studies, thereby enabling a more rigorous assessment of the consistency between the current structural observations and functional evidence.

We thank the reviewer for this important comment. We have substantially revised this section to provide a more detailed description of the structural and functional roles of R24, D25, and R270 reported in previous studies (lines 120–132). Specifically, we added a comparison of previously determined MurJ structures to illustrate the conformational rearrangements of these residues during the transport cycle and revised S6 Fig accordingly. We also discussed previous in vivo crosslinking results involving F22, R24, and R270.

In addition, we revised our interpretation of the lipid-like map to avoid identifying it as a lipid II analog (lines 114-143). Because the map does not contain clearly resolved features corresponding to the hydrophilic headgroup of lipid II, its molecular identity cannot be determined. We therefore now conclude only that a lipid-like molecule may be accommodated in the putative lipid II-binding region even in the SglPP7-bound state.

2. Lines172-188: In the "SglPP7 working model" chapter, the authors' discussion appears somewhat cursory, resulting in insufficient support for the manuscript's central conclusions and a lack of conceptual elevation. The current discussion focuses primarily on comparing similarities and differences between SglPP7 and LysM, while only briefly mentioning the reported SglCJ319. Based on these limited comparisons, the authors arrive at the core conclusion that "Evolutionarily unrelated phage-derived inhibitors target MurJ through distinct molecular strategies, highlighting convergent evolution toward MurJ". However, given the evidence and depth of discussion currently provided, this conclusion appears inadequately substantiated and fails to be entirely convincing. Therefore, I recommend substantial expansion of this chapter to strengthen the logical framework and enhance the persuasiveness of the conclusions. Specifically, the authors could: 1) if feasible, provide more detailed descriptions of the structural and functional similarities and differences among SglCJ319, SglPP7, and LysM, particularly regarding MurJ interaction interfaces, critical residue distributions, and binding modes; 2) if feasible, incorporate 1-2 additional examples of evolutionarily unrelated phage-derived inhibitors and conduct comparative analyses of their structural features to demonstrate the diversity of inhibitory strategies; 3) based on these comparisons, explicitly synthesize how

these distinct strategies collectively point toward convergent evolutionary pressure on MurJ, thereby naturally leading to the manuscript's core conclusion.

We thank the reviewer for this constructive comment. In the revised manuscript, we substantially expanded the discussion of the structural and functional similarities and differences among SglPP7, LysM, and SglCJ3, including their MurJ interaction interfaces, functionally important residues, and binding modes. We also added examples of sequence-divergent phage-derived inhibitors targeting FtsZ and explicitly discussed the current limitations of structural comparison for these proteins. Finally, we revised the discussion to more clearly explain how a shared functional outcome achieved through distinct molecular strategies supports the idea of convergent evolution toward MurJ inhibition (lines186–214).

Minor comments:

1. Lines 54-56 and 72-74: "Although another group reported the structure of a stable mutant MurJ/SglPP7 complex during the preparation of this manuscript" and "Recently, the structure of an N-terminally truncated, Met-Gly-His6-22×GS-tagged SglPP7 (S31A, S39A, S47A)/BRIL-MurJ4-500/Fab-nanobody complex was reported at a resolution of 3.70 Å." Could the authors provide a direct visual comparison in the supplementary materials between the structure reported by the other group and your structure? This would effectively highlight both the similarities and differences between the two studies, as well as emphasize the significance and unique contributions of your work.

We thank the reviewer for this helpful suggestion. We have revised S2 Fig to provide a direct visual comparison between the structures from this study and the previous study. In particular, S2C Fig provides a detailed comparison of the atomic models and cryo-EM maps around the N- and C-terminal regions of SglPP7.

2. Line 85: "Notably, AlphaFold2 (colabfold 1.5.2) and AlphaFold3 failed to predict the correct positioning of SglPP7 on MurJ and showed low confidence in this region, highlighting the importance of the experimentally determined MurJ/SglPP7 complex structure". In this analysis, the authors employed both AlphaFold2 and AlphaFold3 for structural predictions. From a methodological development perspective, AlphaFold3 is a significantly improved version of AlphaFold2, with generally better accuracy and confidence for predicting protein-protein interactions and complex structures. Consequently, given the availability of AlphaFold3 predictions, the additional presentation of AlphaFold2 results offers limited scientific value and may potentially confuse readers regarding the significance of the "prediction failure" conclusion. I recommend that the authors retain only the AlphaFold3 prediction analysis.

We thank the reviewer for this suggestion. We removed the AlphaFold2 predictions and retained only the AlphaFold3 analysis in the revised manuscript. The corresponding text, S3 Fig, and its legend have been revised accordingly (lines 82–89).

3. Line 88: "all AlphaFold MurJ models shown in Supplementary Fig. 3 adopt an inward-facing conformation, in contrast to the outward-facing conformation observed in the experimental structures". This observation deserves more detailed discussion. Given the substantial discrepancies between the AlphaFold-predicted conformations and the experimentally determined complex structure, I recommend that the authors provide speculation or discussion regarding the aforementioned possibilities.

We thank the reviewer for this suggestion. We have added a discussion noting that the inward-facing AlphaFold3 predictions may reflect the predominance of inward-facing MurJ structures in available structural data (lines 82–89).

4. Line 92: "The N-terminal region of SglPP7 (residues 1-22) showed a partial cryo-EM map but could not be reliably modeled, suggesting that it is highly flexible." The authors mention that they obtained only a partial cryo-EM map of the N-terminal region of SglPP7, potentially due to its highly flexible, yet this density map is neither presented in Figure 1 nor referenced elsewhere in the figures.

We thank the reviewer for this comment. We added views of the cryo-EM map around the N-terminal region of SglPP7 in S1A Fig and S2C Fig, and cited these figures in the revised manuscript (lines 67–70, 79–81).

5. Lines 127-137: To evaluate how SglPP7 binding affects the structural dynamics of MurJ, authors performed MD simulations in a membrane environment, both in the presence and absence of SglPP7. This

is an excellent study. However, the analysis of the similarities and differences between the two could be more detailed.
We thank the reviewer for this helpful suggestion. To provide a more detailed comparison between MurJ alone and the MurJ/SglPP7 complex, we performed additional analyses of the MD trajectories. Specifically, we compared the overall conformations at 120 ns and quantified local structural changes by monitoring the Cα–Cα distances between L247–Y266 and G360–G415 in all three independent simulations. We added these analyses to Figure 3C and D and revised the corresponding text to describe both the similarities and differences between the two conditions in greater detail (lines 154–163).

6. Line 185: "when considering the recently reported SglCJ319, which represents another cavity-occupying inhibitor". Please add the structural comparison among SglCJ319, SglPP7 and LysM in the Supplementary Material to better support the proposed theory "Evolutionarily unrelated phage-derived inhibitors target MurJ through distinct molecular strategies, highlighting convergent evolution toward MurJ".
We thank the reviewer for this helpful suggestion. We added a structural comparison among SglPP7, LysM, and SglCJ3 to S7 Fig and S8 Fig, and expanded the corresponding discussion in the revised manuscript (lines 165–214). We also revised the description of the SglCJ3 binding mode based on this comparison.

7. Please provide a more detailed description in the legend for Figure 1A.
We thank the reviewer for this suggestion. We have expanded the legend for Figure 1A to provide a more detailed description (lines 384–389).

8. Please label the protein scale in Figure 1E.
We thank the reviewer for this suggestion. We have added a 10 Å scale bar to the corresponding panel, which is now shown as Figure 1F in the revised manuscript.

9. Please check for consistency in capitalization throughout the Figures; for instance, "sglPP7" in Figure 1B should be corrected.
We thank the reviewer for pointing this out. We checked the capitalization throughout the figures. In Figure 1B, "*sglPP7*" refers to the gene name and is therefore correctly written in lowercase italics. We have ensured that gene and protein names are used consistently throughout the figures.

10. Light-colored numbers in the Figures (e.g., Figure 2, S6, S7) should be bolded or outlined with a dark border to improve accessibility for readers with visual impairments.
We thank the reviewer for this suggestion. We changed the light-colored numbers to darker bold text in Figures 2, S6 Fig, and S7 Fig to improve their visibility.

Reviewer #2:
Hosoda et al present a cryo-electron microscopy structure of the E. coli lipid II flippase MurJ in complex with the phage lysis protein SGLPP7, obtained in nanodiscs, as well some biological experiments and molecular dynamics results.
Overall, this is an interesting and well-written paper, and the conclusions are appropriate for the obtained results. The authors make a good case why it is important to still have this structure in addition to a previous structure from another group that used a truncated SGLPP7 and that was not obtained in lipid nanodiscs.
My main comment is that, based on the validation report, I would suggest the authors to have another look at the modelling given the rather low scores for the Ramachandran of SGLPP7 and the sidechains of MurJ. Also, with the final version the authors should share the coordinates and map to the reviewers.
We thank the reviewer for this important comment. We re-examined the cryo-EM data processing, regenerated the final cryo-EM map, and further refined the atomic model based on the validation report, with particular attention to the Ramachandran geometry of SglPP7 and the side-chain conformations of MurJ. The updated cryo-EM map and coordinate file are provided with the revised submission.

Other comments:

L 92: "The N-terminal region of SglPP7 (residues 1-22) showed a partial cryo-EM map but": replace with "The cryo-EM density for the N-terminal region of SglPP7 (residues 1-22) was very poor" or similar

We thank the reviewer for this suggestion. We revised the sentence to state that the cryo-EM map around the N-terminal region of SglPP7 was very poor and insufficient for reliable atomic modeling (lines 91–94).

L186: "Furthermore, when considering the recently 186 reported SglCJ319, which represents" I assume this is a typo, and the authors mean SglCJ3 and want to reference reference 19? If so, edit the formatting, if it is another phage it needs to be referenced and introduced.

We thank the reviewer for pointing this out. “SglCJ319” resulted from the reference number being placed immediately after “SglCJ3.” Since we substantially revised the paragraph describing the structural comparison, the original sentence is no longer present in the revised manuscript. We have also ensured that reference numbers are clearly separated from protein names throughout the revised manuscript.

L190: There is no conclusion paragraph, the authors should include one

We thank the reviewer for this suggestion. We have added a conclusion paragraph at the end of the Results and Discussion section (lines 226–236).

L198: "de novo": should be italicized

We thank the reviewer for pointing this out. We italicized *de novo* in the revised manuscript (lines 244–245).

L234: "column. preequilibrated": remove dot or replace by a comma

Thank you for the suggestion. We removed the incorrect period and revised the sentence (lines 281–283).

L392: "MurJ/SglPP7" is not bold in title unlike the rest of the title, make bold

Thank you for the suggestion. We have corrected the formatting so that “MurJ/SglPP7” is bolded (line 563).

Supplementary figure 1: "(E) Cryo-EM map (displayed at 4.4σ) of SglPPC and its model.": Describe in more detail how it was visualized.

We thank the reviewer for this suggestion. We revised the legend of S1E Fig to describe in more detail how the cryo-EM map was visualized (lines 568–570).
