# Supplementary material for: Structural basis for inhibition of the lipid flippase MurJ by phage lysis protein SglPP7 suggests convergent evolution with other Sgl peptides
Source: PLoS Biol. 2026 Oct 27;24(10):e3004039. doi: 10.1371/journal.pbio.3004039 (PMC13712586; doi:10.1371/journal.pbio.3004039)
Supplement: Supplementary file 14 [file pbio.3004039.s017.pdf]

Response to the Reviewers

Reviewer #1:

The authors have addressed all my questions, and I have no further questions.

We thank the reviewer for the constructive comments, which have helped us improve our interpretation of the results.

Reviewer #2:

The authors addressed all my queries. Most of the cryo-EM map is of high quality, and the model appears well built. One final minor point came up during inspection of the methods, which the authors can hopefully address easily. In their sample preparation the authors set out to reconstitute their detergent-solubilized sample into nanodiscs, but they do not unambiguously show that the final sample is indeed reconstitute into nanodiscs. In particular, LMNG, which is in their purification buffer, has a low koff. The authors performed a size exclusion after reconsititution into nanodiscs, so presumably they should have SEC profiles and SDS PAGE gels showing co-elution of MSP1D1 and their complex.

We thank the reviewer for this comment. To provide evidence for nanodisc reconstitution, we have added the SEC profile and corresponding SDS–PAGE analysis to S1 Fig., showing co-elution of MSP1D1 and the MurJ/SglPP7 complex. In addition, the cryo-EM map contains density consistent with the MSP scaffold, which is indicated in S1 Fig. These observations support successful reconstitution of the complex into nanodiscs.
