# Supplementary material for: Structural basis for inhibition of the lipid flippase MurJ by phage lysis protein SglPP7 suggests convergent evolution with other Sgl peptides
Source: PLoS Biol. 2026 Oct 27;24(10):e3004039. doi: 10.1371/journal.pbio.3004039 (PMC13712586; doi:10.1371/journal.pbio.3004039)
Supplement: Peer review history

# Editor report: Decision Letter 0

**DOI:** 10.1371/journal.pbio.3004039.r001

**Authors:** Richard Hodge

17 Apr 2026

Dear Dr Tsukazaki,

Thank you for submitting your manuscript entitled "Structural basis for MurJ inhibition by phage lysis protein SglPP7 suggesting convergent evolution" for consideration as a Research Article by PLOS Biology. Please accept my apologies for the delay in getting back to you as we consulted with an academic editor about your submission.

Your manuscript has now been evaluated by the PLOS Biology editorial staff, as well as by an academic editor with relevant expertise, and I am writing to let you know that we would like to send your submission out for external peer review under our complementary research policy.

IMPORTANT - After discussions with the rest of the editorial team, we would like to consider your manuscript as a Short Report at the journal (https://journals.plos.org/plosbiology/s/what-we-publish#loc-short-reports). Upon resubmission (guidelines below), I would be grateful if you could please tick 'Short Report' as the article type in the dropdown menu of the online submission form.

Before we can send your manuscript to reviewers, we need you to complete your submission by providing the metadata that is required for full assessment. To this end, please login to Editorial Manager where you will find the paper in the 'Submissions Needing Revisions' folder on your homepage. Please click 'Revise Submission' from the Action Links and complete all additional questions in the submission questionnaire.

Once your full submission is complete, your paper will undergo a series of checks in preparation for peer review. After your manuscript has passed the checks it will be sent out for review. To provide the metadata for your submission, please Login to Editorial Manager (https://www.editorialmanager.com/pbiology) within two working days, i.e. by Apr 19 2026 11:59PM.

If your manuscript has been previously peer-reviewed at another journal, PLOS Biology is willing to work with those reviews in order to avoid re-starting the process. Submission of the previous reviews is entirely optional and our ability to use them effectively will depend on the willingness of the previous journal to confirm the content of the reports and share the reviewer identities. Please note that we reserve the right to invite additional reviewers if we consider that additional/independent reviewers are needed, although we aim to avoid this as far as possible. In our experience, working with previous reviews does save time.

If you would like us to consider previous reviewer reports, please edit your cover letter to let us know and include the name of the journal where the work was previously considered and the manuscript ID it was given. In addition, please upload a response to the reviews as a 'Prior Peer Review' file type, which should include the reports in full and a point-by-point reply detailing how you have or plan to address the reviewers' concerns.

During the process of completing your manuscript submission, you will be invited to opt-in to posting your pre-review manuscript as a bioRxiv preprint. Visit http://journals.plos.org/plosbiology/s/preprints for full details. If you consent to posting your current manuscript as a preprint, please upload a single Preprint PDF.

Feel free to email us at plosbiology@plos.org if you have any queries relating to your submission.

Kind regards,

Richard

Richard Hodge, PhD

Senior Editor, PLOS Biology

rhodge@plos.org

PLOS

Empowering researchers to transform science

Carlyle House, Carlyle Road, Cambridge, CB4 3DN, United Kingdom

California (U.S.) corporation #C2354500, based in San Francisco

# Peer review: Decision Letter 1

**DOI:** 10.1371/journal.pbio.3004039.r002

**Authors:** Richard Hodge

18 May 2026

Dear Dr Tsukazaki,

Thank you for your patience while your manuscript "Structural basis for MurJ inhibition by phage lysis protein SglPP7 suggesting convergent evolution" was peer-reviewed at PLOS Biology. Please accept my apologies for the delays that you have experienced during the peer review process. Your manuscript has now been evaluated by the PLOS Biology editors, an Academic Editor with relevant expertise, and by two independent reviewers.

In light of the reviews, which you will find at the end of this email, we would like to invite you to revise the work to thoroughly address the reviewers' reports.

As you will see below, both reviewers are generally positive about your manuscript, but Reviewer #1 asks that the manuscript text is more cautious regarding the interpretation of the data and raises concerns with the strength of the evidence supporting the hypothesis of convergent evolution. To address this, the reviewer requests that additional comparative analyses of the structural and functional similarities/differences between SglCJ319, SglPP7, and LysM are provided. In addition, Reviewer #2 requests that the cryo-EM maps and coordinates are provided to confirm that there are no major errors in the modelling of the SglPP7 component given the low validation scores.

Given the extent of revision needed, we cannot make a decision about publication until we have seen the revised manuscript and your response to the reviewers' comments. Your revised manuscript is likely to be sent for further evaluation by all or a subset of the reviewers.

In addition to these revisions, you will need to complete some formatting changes, which you will receive in a follow up email. A member of our team will be in touch with a set of requests shortly.

We expect to receive your revised manuscript within 3 months. Please email us (plosbiology@plos.org) if you have any questions or concerns, or would like to request an extension.

At this stage, your manuscript remains formally under active consideration at our journal; please notify us by email if you do not intend to submit a revision so that we may withdraw it.

**IMPORTANT - SUBMITTING YOUR REVISION**

Your revisions should address the specific points made by each reviewer. Please submit the following files along with your revised manuscript:

1. A 'Response to Reviewers' file - this should detail your responses to the editorial requests, present a point-by-point response to all of the reviewers' comments, and indicate the changes made to the manuscript.

*NOTE: In your point-by-point response to the reviewers, please provide the full context of each review. Do not selectively quote paragraphs or sentences to reply to. The entire set of reviewer comments should be present in full and each specific point should be responded to individually, point by point.

You should also cite any additional relevant literature that has been published since the original submission and mention any additional citations in your response.

2. In addition to a clean copy of the manuscript, please also upload a 'track-changes' version of your manuscript that specifies the edits made. This should be uploaded as a "Revised Article with Changes Highlighted" file type.

*Re-submission Checklist*

When you are ready to resubmit your revised manuscript, please refer to this re-submission checklist: https://plos.io/Biology_Checklist

To submit a revised version of your manuscript, please go to https://www.editorialmanager.com/pbiology/ and log in as an Author. Click the link labelled 'Submissions Needing Revision' where you will find your submission record.

Please make sure to read the following important policies and guidelines while preparing your revision:

*Published Peer Review*

Please note while forming your response, if your article is accepted, you may have the opportunity to make the peer review history publicly available. The record will include editor decision letters (with reviews) and your responses to reviewer comments. If eligible, we will contact you to opt in or out. Please see here for more details:

https://blogs.plos.org/plos/2019/05/plos-journals-now-open-for-published-peer-review/

*PLOS Data Policy*

Please note that as a condition of publication PLOS' data policy (http://journals.plos.org/plosbiology/s/data-availability) requires that you make available all data used to draw the conclusions arrived at in your manuscript. If you have not already done so, you must include any data used in your manuscript either in appropriate repositories, within the body of the manuscript, or as supporting information (N.B. this includes any numerical values that were used to generate graphs, histograms etc.). For an example see here: http://www.plosbiology.org/article/info%3Adoi%2F10.1371%2Fjournal.pbio.1001908#s5

*Blot and Gel Data Policy*

We require the original, uncropped and minimally adjusted images supporting all blot and gel results reported in an article's figures or Supporting Information files. We will require these files before a manuscript can be accepted so please prepare them now, if you have not already uploaded them. Please carefully read our guidelines for how to prepare and upload this data: https://journals.plos.org/plosbiology/s/figures#loc-blot-and-gel-reporting-requirements

*Protocols deposition*

To enhance the reproducibility of your results, we recommend that if applicable you deposit your laboratory protocols in protocols.io, where a protocol can be assigned its own identifier (DOI) such that it can be cited independently in the future. Additionally, PLOS ONE offers an option for publishing peer-reviewed Lab Protocol articles, which describe protocols hosted on protocols.io. Read more information on sharing protocols at https://plos.org/protocols?utm_medium=editorial-email&utm_source=authorletters&utm_campaign=protocols

Thank you again for your submission to our journal. We hope that our editorial process has been constructive thus far, and we welcome your feedback at any time. Please don't hesitate to contact us if you have any questions or comments.

Best regards,

Richard

Richard Hodge, PhD

Senior Editor, PLOS Biology

rhodge@plos.org

------------------------------------

REVIEWS:

Reviewer #1: Hosoda et al. present the cryo-EM structure of the complex between the essential E. coli lipid II flippase MurJ and a phage lysis protein, SglPP7. The authors provide structural, extensive mutational, and molecular dynamics (MD) simulation analyses between MurJ and SglPP7, revealing that SglPP7 binds along the intramembrane cleft of MurJ and inserts its C-terminal tail into the internal cavity, locking MurJ in an outward-facing conformation. Of particular interest is the comparison of SglPP7 with LysM (SglM), a previously characterized MurJ-targeting lysis protein. The study shows that SglPP7 and LysM employ distinct strategies to inhibit MurJ. These findings contribute to the advancement of our understanding of the evolution of unrelated phages, leading to the convergent evolution of distinct strategies to inhibit this essential host protein. The present study is comprehensive and represents an important contribution to the field, offering high-resolution insights into the elusive interactional environment inside the intramembrane. Nevertheless, the following recommendations are hereby made for the improvement of the manuscript.

Major comments:

1.Lines 115-125: In the "A lipid-like molecule between TM1, TM8, and TM14 of MurJ" chapter, the authors primarily relied on analogical reasoning, interpreting an unassigned density inside the MurJ-SglPP7 complex as potentially mimicking lipid II binding. In their structure, the lipid-like feature is located near residues F22, R24, and R270. Furthermore, previous biochemical and structural studies have shown that R24, D25, and R270 are essential for cell viability and have been proposed to form the binding site for the phosphate group of lipid II. Although the similarity between these regions supports the authors' reasoning, notable differences remain in the binding sites; for protein-ligand interactions, even minor discrepancies can induce significant functional variations. Consequently, I remain cautious regarding the authors' direct interpretation of this density as a lipid II analog. I recommend that the authors provide a more detailed description of the specific roles and binding characteristics of R24, D25, and R270 as reported in published studies, thereby enabling a more rigorous assessment of the consistency between the current structural observations and functional evidence.

2.Lines172-188: In the "SglPP7 working model" chapter, the authors' discussion appears somewhat cursory, resulting in insufficient support for the manuscript's central conclusions and a lack of conceptual elevation. The current discussion focuses primarily on comparing similarities and differences between SglPP7 and LysM, while only briefly mentioning the reported SglCJ319. Based on these limited comparisons, the authors arrive at the core conclusion that "Evolutionarily unrelated phage-derived inhibitors target MurJ through distinct molecular strategies, highlighting convergent evolution toward MurJ". However, given the evidence and depth of discussion currently provided, this conclusion appears inadequately substantiated and fails to be entirely convincing. Therefore, I recommend substantial expansion of this chapter to strengthen the logical framework and enhance the persuasiveness of the conclusions. Specifically, the authors could: 1) if feasible, provide more detailed descriptions of the structural and functional similarities and differences among SglCJ319, SglPP7, and LysM, particularly regarding MurJ interaction interfaces, critical residue distributions, and binding modes; 2) if feasible, incorporate 1-2 additional examples of evolutionarily unrelated phage-derived inhibitors and conduct comparative analyses of their structural features to demonstrate the diversity of inhibitory strategies; 3) based on these comparisons, explicitly synthesize how these distinct strategies collectively point toward convergent evolutionary pressure on MurJ, thereby naturally leading to the manuscript's core conclusion.

Minor comments:

1.Lines 54-56 and 72-74: "Although another group reported the structure of a stable mutant MurJ/SglPP7 complex during the preparation of this manuscript" and "Recently, the structure of an N-terminally truncated, Met-Gly-His6-22×GS-tagged SglPP7 (S31A, S39A, S47A)/BRIL-MurJ4-500/Fab-nanobody complex was reported at a resolution of 3.70 Å." Could the authors provide a direct visual comparison in the supplementary materials between the structure reported by the other group and your structure? This would effectively highlight both the similarities and differences between the two studies, as well as emphasize the significance and unique contributions of your work.

2.Line 85: "Notably, AlphaFold2 (colabfold 1.5.2) and AlphaFold3 failed to predict the correct positioning of SglPP7 on MurJ and showed low confidence in this region, highlighting the importance of the experimentally determined MurJ/SglPP7 complex structure". In this analysis, the authors employed both AlphaFold2 and AlphaFold3 for structural predictions. From a methodological development perspective, AlphaFold3 is a significantly improved version of AlphaFold2, with generally better accuracy and confidence for predicting protein-protein interactions and complex structures. Consequently, given the availability of AlphaFold3 predictions, the additional presentation of AlphaFold2 results offers limited scientific value and may potentially confuse readers regarding the significance of the "prediction failure" conclusion. I recommend that the authors retain only the AlphaFold3 prediction analysis.

3.Line 88: "all AlphaFold MurJ models shown in Supplementary Fig. 3 adopt an inward-facing conformation, in contrast to the outward-facing conformation observed in the experimental structures". This observation deserves more detailed discussion. Given the substantial discrepancies between the AlphaFold-predicted conformations and the experimentally determined complex structure, I recommend that the authors provide speculation or discussion regarding the aforementioned possibilities.

4.Line 92: "The N-terminal region of SglPP7 (residues 1-22) showed a partial cryo-EM map but could not be reliably modeled, suggesting that it is highly flexible." The authors mention that they obtained only a partial cryo-EM map of the N-terminal region of SglPP7, potentially due to its highly flexible, yet this density map is neither presented in Figure 1 nor referenced elsewhere in the figures.

5.Lines 127-137: To evaluate how SglPP7 binding affects the structural dynamics of MurJ, authors performed MD simulations in a membrane environment, both in the presence and absence of SglPP7. This is an excellent study. However, the analysis of the similarities and differences between the two could be more detailed.

6.Line 185: "when considering the recently reported SglCJ319, which represents another cavity-occupying inhibitor". Please add the structural comparison among SglCJ319, SglPP7 and LysM in the Supplementary Material to better support the proposed theory "Evolutionarily unrelated phage-derived inhibitors target MurJ through distinct molecular strategies, highlighting convergent evolution toward MurJ".

7.Please provide a more detailed description in the legend for Figure 1A.

8.Please label the protein scale in Figure 1E.

9.Please check for consistency in capitalization throughout the Figures; for instance, "sglPP7" in Figure 1B should be corrected.

10.Light-colored numbers in the Figures (e.g., Figure 2, S6, S7) should be bolded or outlined with a dark border to improve accessibility for readers with visual impairments.

Reviewer #2: Hosoda et al present a cryo-electron microscopy structure of the E. coli lipid II flippase MurJ in complex with the phage lysis protein SGLPP7, obtained in nanodiscs, as well some biological experiments and molecular dynamics results.

Overall, this is an interesting and well-written paper, and the conclusions are appropriate for the obtained results. The authors make a good case why it is important to still have this structure in addition to a previous structure from another group that used a truncated SGLPP7 and that was not obtained in lipid nanodiscs.

My main comment is that, based on the validation report, Iwould suggest the authors to have another look at the modelling given the rather low scores for the Ramachandran of SGLPP7 and the sidechains of MurJ. Also, with the final version the authors should share the coordinates and map to the reviewers.

Other comments:

L 92: "The N-terminal region of SglPP7 (residues 1-22) showed a partial cryo-EM map but": replace with "The cryo-EM density for the N-terminal region of SglPP7 (residues 1-22) was very poor" or similar

L186: "Furthermore, when considering the recently 186 reported SglCJ319, which represents" I assume this is a typo, and the authors mean SglCJ3 and want to reference reference 19? If so, edit the formatting, if it is another phage it needs to be referenced and introduced.

L190: There is no conclusion paragraph, the authors should include one

L198: "de novo": should be italicized

L234: "column. preequilibrated": remove dot or replace by a comma

L392: "MurJ/SglPP7" is not bold in title unlike the rest of the title, make bold

Supplementary figure 1: "(E) Cryo-EM map (displayed at 4.4σ) of SglPPC and its model.": Describe in more detail how it was visualized.

# Author comment: Author response to Decision Letter 2

**DOI:** 10.1371/journal.pbio.3004039.r003

24 Aug 2026

Attachment Submitted filename: Response to the Reviewers_commentsONLY.pdf

# Peer review: Decision Letter 2

**DOI:** 10.1371/journal.pbio.3004039.r004

**Authors:** Richard Hodge

15 Sep 2026

Dear Dr Tsukazaki,

Thank you for your patience while we considered your revised manuscript "Structural basis for MurJ inhibition by phage lysis protein SglPP7 suggesting convergent evolution" for publication as a Short Report at PLOS Biology. This revised version of your manuscript has been evaluated by the PLOS Biology editors, the Academic Editor and the original reviewers.

Based on the reviews, I am pleased to say that we are likely to accept this manuscript for publication, provided you satisfactorily address the remaining points raised by Reviewer #2. In addition, please also make sure to address the following data and other policy-related requests that I have provided below (A-E):

(A) Please note that we are currently considering your manuscript as a Short Report, which have a maximum of 4 main figures. I would be grateful if you could please move the schematic diagram in Figure 5 to the supplementary figures or combine Figure 4.

(B) We routinely suggest changes to titles to ensure maximum accessibility for a broad, non-specialist readership. In this case, we suggest a minor edit to the title, as follows, to provide some additional information. Please ensure you change both the manuscript file and the online submission system, as they need to match for final acceptance:

"Structural basis for inhibition of the lipid flippase MurJ by phage lysis protein SglPP7 suggests convergent evolution with other Sgl peptides"

(C) Thank you for already depositing the structural data in the PDB (9X2N) and EMDB (EMD-66480) databases. However, we note that the structures are currently on hold for release. At this stage, we ask that you please ensure that the structural data is made publicly available before publication.

(D) Please also ensure that each of the relevant figure legends in your manuscript include information on *WHERE THE UNDERLYING DATA CAN BE FOUND*, and ensure your supplemental data file/s has a legend.

(E) Per journal policy, if you have generated any custom code during the course of this investigation, please make it available without restrictions. Please ensure that the code is sufficiently well documented and reusable, and that your Data Statement in the Editorial Manager submission system accurately describes where your code can be found. More information on our Code Policy, what and how to share can be found here: https://journals.plos.org/plosbiology/s/code-availability

Please note that we cannot accept sole deposition of code in GitHub, as this could be changed after publication. However, you can archive this version of your publicly available GitHub code to Zenodo. Once you do this, it will generate a DOI number, which you will need to provide in the Data Accessibility Statement (you are welcome to also provide the GitHub access information). See the process for doing this here: https://docs.github.com/en/repositories/archiving-a-github-repository/referencing-and-citing-content

------------------------------------------------------------------------

As you address these items, please take this last chance to review your reference list to ensure that it is complete and correct. If you have cited papers that have been retracted, please include the rationale for doing so in the manuscript text, or remove these references and replace them with relevant current references. Any changes to the reference list should be mentioned in the cover letter that accompanies your revised manuscript.

In addition to these revisions, you may need to complete some formatting changes, which you will receive in a follow up email. A member of our team will be in touch with a set of requests shortly. If you do not receive a separate email within a few days, please assume that checks have been completed, and no additional changes are required.

We expect to receive your revised manuscript within two weeks.

To submit your revision, please go to https://www.editorialmanager.com/pbiology/ and log in as an Author. Click the link labelled 'Submissions Needing Revision' to find your submission record. Your revised submission must include the following:

- a cover letter that should detail your responses to any editorial requests, if applicable, and whether changes have been made to the reference list

- a Response to Reviewers file that provides a detailed response to the reviewers' comments (if applicable, if not applicable please do not delete your existing 'Response to Reviewers' file.)

- a track-changes file indicating any changes that you have made to the manuscript.

NOTE: If Supporting Information files are included with your article, note that these are not copyedited and will be published as they are submitted. Please ensure that these files are legible and of high quality (at least 300 dpi) in an easily accessible file format. For this reason, please be aware that any references listed in an SI file will not be indexed. For more information, see our Supporting Information guidelines:

https://journals.plos.org/plosbiology/s/supporting-information

*Published Peer Review History*

Please note that you may have the opportunity to make the peer review history publicly available. The record will include editor decision letters (with reviews) and your responses to reviewer comments. If eligible, we will contact you to opt in or out. Please see here for more details:

https://plos.org/published-peer-review-history/

*Press*

Should you, your institution's press office or the journal office choose to press release your paper, please ensure you have opted out of Early Article Posting on the submission form. We ask that you notify us as soon as possible if you or your institution is planning to press release the article.

*Protocols deposition*

To enhance the reproducibility of your results, we recommend that if applicable you deposit your laboratory protocols in protocols.io, where a protocol can be assigned its own identifier (DOI) such that it can be cited independently in the future. Additionally, PLOS ONE offers an option for publishing peer-reviewed Lab Protocol articles, which describe protocols hosted on protocols.io. Read more information on sharing protocols at https://plos.org/protocols?utm_medium=editorial-email&utm_source=authorletters&utm_campaign=protocols

Please do not hesitate to contact me should you have any questions.

Best wishes,

Richard

Richard Hodge, PhD

Senior Editor, PLOS Biology

rhodge@plos.org

------------------------------------------------------------------------

Reviewer remarks:

Reviewer #1: The authors have addressed all my questions, and I have no further questions.

Reviewer #2: The authors addressed all my queries. Most of the cryo-EM map is of high quality, and the model appears well built.

One final minor point came up during inspection of the methods, which the authors can hopefully address easily. In their sample preparation the authors set out to reconstitute their detergent-solubilized sample into nanodiscs, but they do not unambiguously show that the final sample is indeed reconstitute into nanodiscs. In particular, LMNG, which is in their purification buffer, has a low koff. The authors performed a size exclusion after reconsititution into nanodiscs, so presumably they should have SEC profiles and SDS PAGE gels showing co-elution of MSP1D1 and their complex.

# Author comment: Author response to Decision Letter 3

**DOI:** 10.1371/journal.pbio.3004039.r005

18 Sep 2026

Attachment Submitted filename: Response to the Reviewers_20260918_v2.pdf

# Editor report: Decision Letter 3

**DOI:** 10.1371/journal.pbio.3004039.r006

**Authors:** Richard Hodge

21 Sep 2026

Dear Tomoya,

On behalf of my colleagues and the Academic Editor, David Bhella, I am pleased to say that we can accept your manuscript for publication, provided you address any remaining formatting and reporting issues. These will be detailed in an email you should receive within 2-3 business days from our colleagues in the journal operations team; no action is required from you until then. Please note that we will not be able to formally accept your manuscript and schedule it for publication until you have completed any requested changes.

Please take a minute to log into Editorial Manager at http://www.editorialmanager.com/pbiology/, click the "Update My Information" link at the top of the page, and update your user information to ensure an efficient production process.

PRESS

We frequently collaborate with press offices. If your institution or institutions have a press office, please notify them about your upcoming paper at this point, to enable them to help maximise its impact. If the press office is planning to promote your findings, we would be grateful if they could coordinate with biologypress@plos.org. If you have previously opted in to the early version process, we ask that you notify us immediately of any press plans so that we may opt out on your behalf.

We also ask that you take this opportunity to read our Embargo Policy regarding the discussion, promotion and media coverage of work that is yet to be published by PLOS. As your manuscript is not yet published, it is bound by the conditions of our Embargo Policy. Please be aware that this policy is in place both to ensure that any press coverage of your article is fully substantiated and to provide a direct link between such coverage and the published work. For full details of our Embargo Policy, please visit http://www.plos.org/about/media-inquiries/embargo-policy/.

Thank you again for choosing PLOS Biology for publication and supporting Open Access publishing. We look forward to publishing your study.

Best wishes,

Richard

Richard Hodge, PhD

Senior Editor, PLOS Biology

rhodge@plos.org

PLOS

Empowering researchers to transform science

Carlyle House, Carlyle Road, Cambridge, CB4 3DN, United Kingdom

California (U.S.) corporation #C2354500, based in San Francisco
